# A work flow to build and validate patient specific left atrium electrophysiology models from catheter measurements

**DOI:** 10.1016/j.media.2018.04.005

**Published:** 2018-07

**Authors:** Cesare Corrado, Steven Williams, Rashed Karim, Gernot Plank, Mark O’Neill, Steven Niederer

**Affiliations:** a Division of Imaging Sciences & Biomedical Engineering, King’s College London, London SE17EH, United Kingdom; b Department of Biophysics, Medical University of Graz, Neue Stiftingtalstraße 6/IV, 8010 Graz, Austria

**Keywords:** Atria, Patient specific, Biophysical modelling, Validation

## Abstract

Biophysical models of the atrium provide a physically constrained framework for describing the current state of an atrium and allow predictions of how that atrium will respond to therapy. We propose a work flow to simulate patient specific electrophysiological heterogeneity from clinical data and validate the resulting biophysical models. In 7 patients, we recorded the atrial anatomy with an electroanatomical mapping system (St Jude Velocity); we then applied an S1–S2 electrical stimulation protocol from the coronary sinus (CS) and the high right atrium (HRA) whilst recording the activation patterns using a PentaRay catheter with 10 bipolar electrodes at 12 ± 2 sites across the atrium. Using only the activation times measured with a PentaRay catheter and caused by a stimulus applied in the CS with a remote catheter we fitted the four parameters for a modified Mitchell–Schaeffer model and the tissue conductivity to the recorded local conduction velocity restitution curve and estimated local effective refractory period. Model parameters were then interpolated across each atrium. The fitted model recapitulated the S1–S2 activation times for CS pacing giving a correlation ranging between 0.81 and 0.98. The model was validated by comparing simulated activations times with the independently recorded HRA pacing S1–S2 activation times, giving a correlation ranging between 0.65 and 0.96. The resulting work flow provides the first validated cohort of models that capture clinically measured patient specific electrophysiological heterogeneity.

## Highlights

• Locally personalised atrial electrophysiology.
• Predictive simulations.
• Catheter measurements.
• Clinical time scale.
• Atrial fibrillation.

## Graphical abstract

**Figure:**
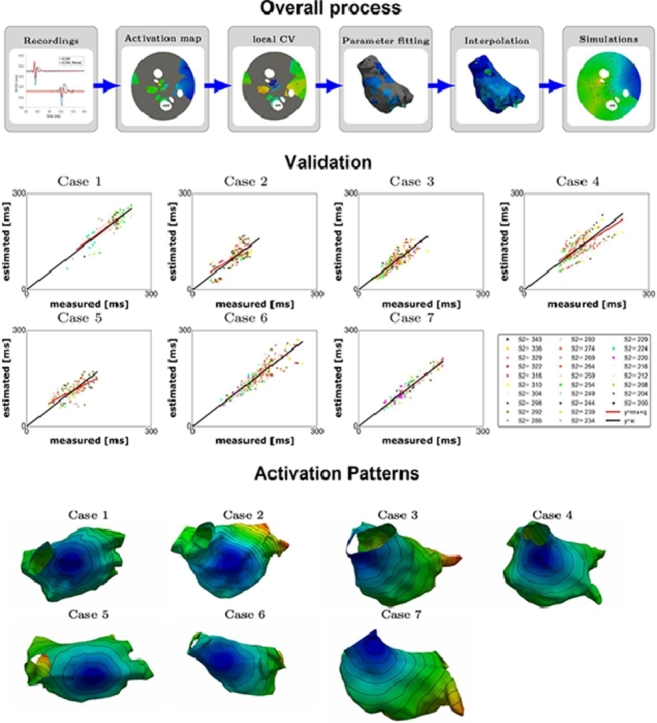

## 1 Introduction

Atrial fibrillation (AF) is a supra-ventricular tachyarrhythmia that is characterised by an uncoordinated activation of the atrial tissue (Skanes, Mandapati, Berenfeld, Davidenko, Jalife, 1998, Konings, Kirchhof, Smeets, Wellens, Penn, Allessie, 1994), with a consequent deterioration of mechanical function, Reant et al. (2005). AF is associated with an increased incidence of other cardiovascular diseases, stroke and premature death, Chugh et al. (2013). In drug refractory patients, AF is commonly treated by radio-frequency ablation (Haïssaguerre, et al., 2000, Oral, et al., 2002). In many patients the mechanisms underpinning AF are unknown and there are no consensus guidelines for treating all patients (Marchlinski, 2008, Cosío, 2011), with many patients requiring multiple procedures to achieve sinus rhythm (Cappato et al., 2005).

Local tissue properties, identified by fractionated electrograms (Nademanee et al., 2004), and a heterogeneous atrial substrate, O’Neill et al. (2006) have been proposed to play a significant role in initiating and sustaining AF. However, measuring these patient characteristics and linking them to AF sustenance and treatment remain challenging.

Patient specific models have been proposed as a novel approach to combine anatomical and electrical data to identify the mechanisms underpinning AF in individual patients and to predict the optimal therapy on a case by case basis, Colli Franzone et al. (2006), Kneller et al. (2002), Aslanidi et al. (2011). These frameworks brought to light the importance of including the electrophysiological heterogeneity of the substrate for predicting activation patterns in the atrium, Kneller et al. (2002) and Aslanidi et al. (2011), consistent with clinical observations, O’Neill et al. (2006).

Recent advances in medical imaging have resulted in models that capture individual anatomy and regions of fibrotic tissue (McDowell et al., 2012) but no modelling framework has captured individual electrophysiological properties and the distribution of these properties across the atrium. Data assimilation techniques have been proposed to infer heterogeneous model parameters in the ventricles directly from clinical data in individual patients, Corrado et al. (2015). These techniques however relied upon activation and repolarisation data, which are not available from conventional atrial electrocardiogram recordings, and involve a fitting process that is computationally expensive.

Previously we have proposed a robust and rapid pacing protocol and a model-fitting algorithm for personalising atrial electrophysiological models, Corrado et al. (2016a). We have developed a refined cell model (Corrado and Niederer, 2016) that does not exhibit pacemaker behaviour, removing the need to test for ectopic beats in model fitting and demonstrated that this model can be robustly used to infer local material properties from clinical data, Corrado et al. (2016b).

In this paper we present a work-flow that applies our proposed local electrophysiological fitting approach to several sites of the left atrium and then generates locally personalised electrophysiology models of atrial electrophysiology that span the whole left atrium. Previous studies (Krueger et al., 2013) built patient-specific models of the atrial anatomy and personalised the electrophysiology using one catheter location only. We then validate the work-flow with 7 clinical cases by comparing the data activation times from clinical experiments with predicted activation times generated from simulations. The predicted and the clinical data showed a correlation ranging between 0.65 and 0.96 when planar propagation was considered: this work-flow paves the way to the personalisation of the procedure treating AF as it enables predictions on the possible outcomes.

## 2 Method

**Fig. 1:**
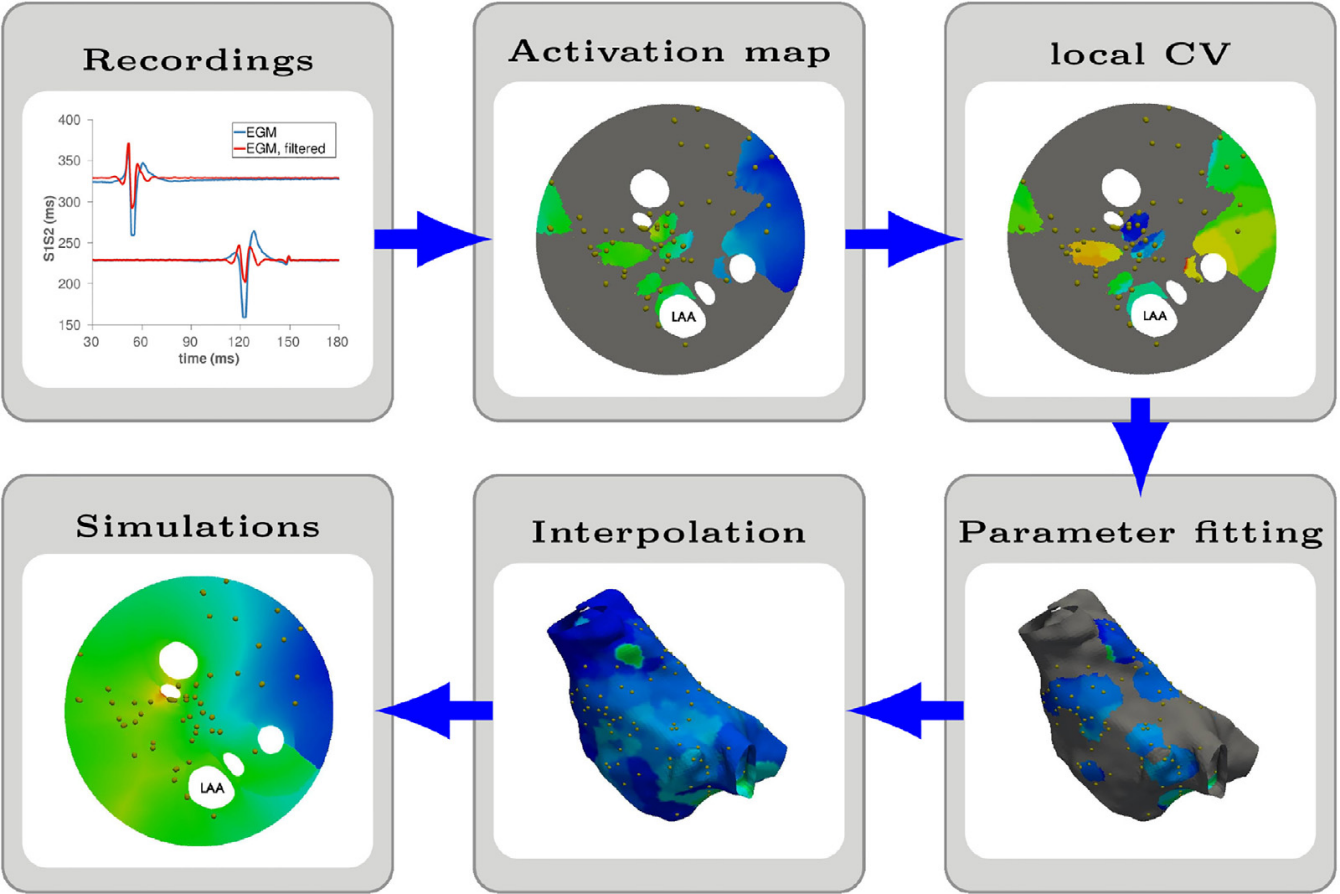
Schematic procedure followed to generate a computational model of the human atrium. The activation map, the local CV and the simulated LATs are depicted on an unfolded geometry, Karim et al. (2014); the gold dots corresponds to the location where bipolar electrograms were recorded.

The atrial model is made in six steps as depicted in Fig. 1. First, we record clinical data (Section 2.1). Second, we process the electro-anatomical mapping data and the corresponding electrograms (EGM) to identify the local activation times (LATs) for each pacing protocol (Section 2.2) and estimate the local effective refractory period (ERP) and the local conduction velocity (CV) at each coupling interval (Section 2.3). The cell and local tissue activation model are described in Section 2.4. Third, we fit the cell model parameters and the tissue conductivity to the ERP and the local CV restitution (Section 2.5). The proposed modelling framework for simulating the left atrium is described in Section 2.6. Next, we interpolate and extrapolate the local cell model and tissue parameters across the regions of the atrium where no measurements are available (Section 2.7). In step five a simulation of the atrial activation and repolarisation is implemented (Section 2.8) for an external stimulus on the left atrium located to minimise the activation error at the largest coupling interval (Section 2.9). Finally, we validate the computational model (Section 2.10) by comparing the measured LATs, with the set of LATs obtained from the numerical simulations of the clinical pacing procedure.

### 2.1 Clinical measurements

Data for this study were collected as part of Williams et al. (2017b). Here we provide a brief summary of the clinical methods.

#### 2.1.1 Patient selection and clinical procedures

In this paper, we considered clinical patients suffering from short, intermittent AF (paroxysmal) and undergoing first time atrial fibrillation ablation. None of the cases presented large regions of low voltage (<  0.3 mV). Ethical approval was granted by the National Research Ethics Service (10/H0802/77) and all participants gave written informed consent for inclusion in the study. The research conformed to the principles described in the Declaration of Helsinki. Patients with ischemic heart disease, cardiac surgery or structural heart disease were excluded. Anti-arrhythmic drugs, including calcium channel blockers, were stopped at least 5 half-lives before ablation. Amiodarone was stopped at least 6 weeks prior to ablation. All clinical procedures were performed under general anaesthesia. Following femoral access and trans-septal puncture, two 8.5 French SR0 long sheaths and a PentaRay mapping catheter (Biosense Webster, CA, 1mm electrode size, 4-4-4mm spacing) were advanced into the LA. Decapole (St Jude Medical, MN) and pentapole (Bard Electrophysiology, MA) catheters were positioned in the coronary sinus and high right atrium, respectively.

#### 2.1.2 Pacing protocol

The pacing protocol was delivered using a custom-built, institutionally-approved stimulator and consisted of a 2-beat drive train ($s_{1}=470\;\mathrm{ms}$) followed by a single premature extra stimulus *s*_2_ < *s*_1_. The *s*_1_*s*_2_ coupling interval was reduced continuously and without operator interference in 2% steps from 343 ms to either 200 ms or loss of capture. All pacing stimuli were delivered at a voltage of at least twice the threshold voltage and with a pulse width of 2 ms. Pacing stimuli were delivered from either the CS or the HRA, while the activation times were recorded in the body of the LA using a PentaRay catheter. The PentaRay catheter was sequentially manoeuvred to multiple sites on the endocardial surface of the LA; bipolar electrograms were recorded throughout in response to complete *s*_1_*s*_2_ pacing trains, delivered from the mid-CS or HRA. Details on the number of the recorded data points for each patient and for each stimulus location is described in the online supplement.

### 2.2 Processing electrograms

We processed the atrial bipolar electrograms from the PentaRay using an in-house developed MATLAB graphical user interface (GUI). This interface reads in the output files from the electroanatomical mapping system (Ensite Velocity St Jude) and registers the positions of the recording electrodes to the mapped atrial geometry. For each bipolar pair of electrodes and for each *s*_1_*s*_2_ coupling interval, we evaluated LATs as the time that the first peak (or valley) on the electrogram trace occurs. On bipolar electrodes, where measurements are available, we evaluated CV using a piecewise linear approach, similar to that described in Cantwell et al. (2015) and summarised below as follows:

1. On the site identified by the electrodes forming the PentaRay catheter, we interpolate the LATs measured at the 10 bipolar electrodes using piecewise-linear polynomia and a Delaunay triangulation.
2. We locally compute the gradient of the interpolated LATs and then we evaluate the modulus of the local CV as the inverse of the modulus of the gradient.
3. On each electrode that captured a signal and for each premature extra stimulus *s*_2_, we identified the local CV as the median of the computed CV values within a circular region of radius R=2.5 cm around the electrode. This radius corresponds to twice of the length of a catheter spline and removes spurious CV values from the fitting.

All calculated fields (LAT and CV at each electrode for each pacing protocol) and the anatomy are then exported to VTK files (Schroeder et al., 2004) providing the input for the model personalisation process.

### 2.3 Generating the input for the fitting algorithm

From the local CV values, described above, we generated local CV restitutions using the PentaRay electrograms at each electrode and for each *s*_1_*s*_2_ pacing protocol when the atrium was paced in the CS. Plots of the measured CV restitutions at representative points of the atria are depicted in the online supplement. We defined CV values to be outside of the physiological range if they were greater than 200 cm/s. We determined this value by first plotting the CV distribution for all the *s*_2_ coupling intervals and all the clinical data considered, then fitting that distribution to a normal distribution and rounding the value that corresponds to the mean+2SD. The distribution in CV is presented in the on-line supplement and is consistent with those reported in Okano et al. (2010) and Fukumoto et al. (2016); Weber et al. (2011) on the left atrium and with those reported in Schilling et al. (2001) for the right atrium. We estimated the local ERP value as the largest *s*_2_ interval that did not produce a local activation at the PentaRay electrode. This value was termed *S*1*S*2_block_ in Williams et al. (2017b). Since the pacing protocol uses a single *s*_1_ value, only one ERP value is estimated at each PentaRay electrode, not a restitution curve.

### 2.4 Modelling the local electrophysiology

We characterised the local electrophysiology with the modified Mitchell–Schaffer (mMS) model presented in Corrado and Niederer (2016). Like the classical Mitchell-Schaeffer (MS) model (Mitchell and Schaeffer, 2003), this model is described by 4 parameters (*τ*_in_, *τ*_out_, *τ*_open_, *τ*_close_), each representing the characteristic time constant of 4 distinct phases of the action potential. Importantly and in contrast to the original MS model, the mMS model does not demonstrate pacemaker behaviour, for any parameter combination.

The chosen cell model is able to capture the measured restitution properties with the smallest number of parameters. The model complexity was selected to reflect the available clinical data. Physiological mechanisms, including cardiac memory and intracellular calcium handling were not recorded and so were not included in the model.

### 2.5 Fitting local electrophysiology parameters

We determined the values of the parameters characterising the tissue electrophysiology by applying the algorithm described in Corrado, Whitaker, Chubb, Williams, Wright, Gill, O’Neill, Niederer, 2016, Corrado, Whitaker, Chubb, Williams, Wright, Gill, O’Neill, Niederer, 2016. This algorithm fits the restitution curves, generated from clinical recordings with the procedure described in Section 2.3, to a set of pre-computed restitutions, obtained by solving a computational model that numerically reproduces the clinical procedure. In this paper, we fit the model parameters to the CV and ERP derived from activation waves measured at the PentaRay electrode locations and caused by a stimulus applied in the CS. We estimated the local ERP value as the largest *s*_2_ interval that did not produce a local activation. Differently from the procedure described in Corrado, Whitaker, Chubb, Williams, Wright, Gill, O’Neill, Niederer, 2016, Corrado, Whitaker, Chubb, Williams, Wright, Gill, O’Neill, Niederer, 2016, in this work we fit one ERP as the pacing protocol has a single *s*_1_, value and not three as used previously to generate a restitution curve. We introduced a regularisation on the space distribution of the parameters by first evaluating the median values of the parameters on the electrodes and then introducing in the procedure described in Corrado, Whitaker, Chubb, Williams, Wright, Gill, O’Neill, Niederer, 2016, Corrado, Whitaker, Chubb, Williams, Wright, Gill, O’Neill, Niederer, 2016 a penalisation term, proportional to the difference between the candidate parameters and the median values. For each clinical case, the measured CV restitutions and the restitution curves obtained with the fitting process, prior to the regularisation step and with the spatial regularisation are depicted in the online supplement.

We obtained the pre-computed restitutions from the solution of the following 1D monodomain mMS electrophysiology problem:

$$\partial_{t}v_{m}=D\partial_{xx}v_{m}+I_{\mathrm{ion}}\left(v_{m},h\right)+I_{\mathrm{app}}$$

where *v*_m_ denotes the trans-membrane potential, *D* denotes the diffusion coefficient, *h* denotes the gate variable, *I*_ion_(*v*_m_, *h*) denotes the reaction term characterising the ionic current and *I*_app_ denotes the externally applied stimulus triggering the depolarisation. We considered a 1D domain with a length L=10cm, stimulated at one end, on a region of length $L_{\mathrm{stim}}=0.5\;\mathrm{mm}$ and following the pacing procedure. We discretised the problem in space with a characteristic mesh size h=200μm; we dealt with non-linarites through the splitting scheme described in Keener and Sneyd (1998); we discretised the cell model with a forward Euler scheme and a time step $dt_{\mathrm{ODE}}=10\;\mu s,$ while we solved the diffusivity problem with a second order Crank–Nicholson scheme and a time step $dt_{\mathrm{PDE}}=100\;\mu s$.

We then computed the extracellular potential by solving the 1D equation of the extracellular potential *ϕ_e_*:

$$\begin{array}{c} \frac{d^{2}}{dx^{2}}\left(v_{m}+2\phi_{e}\right)=0 \end{array}$$

obtained by assuming uniform conductivity in the monodomain model. We assume a mean extracellular potential of zero, as described in the on-line supplement in Corrado et al. (2016a) and thus calculated the bipolar signal is the difference of the extracellular potential evaluated at the two poles forming the electrode. We computed the bipolar signals for two bipolar pairs of electrodes separated by a distance of 2 mm and a distance of 7 mm between the barycentre of the two pairs of electrodes, located in the middle of the domain *L* to minimise boundary effects. Finally we evaluated the CV restitutions. Differently from Corrado, Whitaker, Chubb, Williams, Wright, Gill, O’Neill, Niederer, 2016, Corrado, Whitaker, Chubb, Williams, Wright, Gill, O’Neill, Niederer, 2016, in this paper we created a data set by sweeping on the parameter set defined by the maximum value of the action potential duration, APD_max_, the minimum value of the gate variable on the null-cline, *h*_min_ defined in Corrado and Niederer (2016), the maximum conduction velocity, CV_max_, and the *τ*_in_ and *τ*_open_ ionic parameters; we obtained the original set of parameters characterising the mMS model by inverting the analytical leading order formulations for CV_max_, APD_max_ and *h*_min_. Details on the procedure can be found in the online supplement. Even though leading order approximations contain errors, this procedure presents the following two benefits: first, leading order approximations provide the order of magnitude of the biophysical marker involved and thus improve the building of a bespoke data set; second, since the sweeping is performed on the observed quantities, the sensitivity of the observations on the parameter variations is implicitly obtained.

In this paper, we evaluated a data set of pre-computed restitutions for a total of 580,800 parameter combinations, characterised by the values summarised in Table 1. For the parameter *h*_min_, we chose the candidate values with a step of 0.02 within the interval [0.01, 0.09] and with a step of 0.1 within the interval [0.1, 0.5].

**Table 1:** Parameter values used for building the data set. A set of parameter values ranging from the minimum to the maximum value in increments of the step value is created. The data set of candidate solutions was generated by models with each of the permutations of the Cartesian products of all of the parameter value sets.

	CV_max_ (cm/s)	*τ*_in_ (ms)	*h*_min_ (adim)	*τ*_open_ (ms)	APD_max_ (ms)
Min	10	0.01	[0.01; 0.1]	65	120
Max	300	0.31	[0.09; 0.5]	215	270
Step	10	0.03	[0.02; 0.1]	10	15

Locally, the tissue is characterised by the conductivity and the 4 characteristic time constants of the mMS model, while the gate potential was fixed to a value $v_{\mathrm{gate}}\;=\;0.1$ (dimensionless).

### 2.6 Modelling the left atrium

In this paper, we describe the atrium as a 2D tissue shell. The electrophysiology is modelled using the isotropic monodomain equations, Keener and Sneyd (1998), this reduces the diffusion tensor to a single scalar value. The model of the atrium aimed to capture the level of complexity reflected in the available clinical data and how these data are interpreted by cardiologists. Measurements are recorded solely on the endocardial surface and despite the potential for transmural activation and the complex fibre structure of the atrium to play a role in AF, these effects are not considered (routinely) during clinical procedures and so we do not consider them here. For these reasons we represent the atrium in the simplest model possible, given the available clinical data: an isotropic shell model, with heterogeneous material properties.

### 2.7 Interpolating local parameters across the whole atrium

We determined the heterogeneous material properties from clinical measurements as described in Section 2.5. Measurements were not performed across the entire atrium, leaving areas where no directly measured material properties were available. To infer the parameters across the entire atrial domain, we characterised each geometrical point of the computational mesh produced by the electro anatomical mapping system (St. Jude) by the parameter values estimated at the closest electrodes with respect to the Euclidean distance. We also tested applying a Gaussian filter and a Poisson based interpolation scheme, to estimate the atrial properties on locations where no direct measurements of material properties were available; these different approaches were more complex and produced results that were either equivalent or worse to those obtained with the nearest neighbour criterion. Results are reported in the online supplement. The models of the atrium were defined in the Cardiac Arrhythmias Package (CARP) (Niederer, Mitchell, Smith, Plank, 2011, Vigmond, Hughes, Plank, Leon, 2003, Vigmond, Weber dos Santos, Prassl, Deo, Plank, 2008). In this framework, the conductivities are defined on element groups. In each case, the interpolated and smoothed continuous conductivities were binned into 200 element sets with distinct values. For each clinical case, the distributions of the values of each parameter considered in this paper are depicted in the online supplement.

### 2.8 The computational model

We obtained a smoothed geometric mesh (Niederer et al., 2011b) by smoothing the electro-anatomical mapping system anatomical mesh with the Poisson filter implemented in the MESHLAB library, CNR and then clipping the pulmonary veins and the mitral annulus with the tools provided by the VMTK library, Antiga et al. (2008). With the same library, we generated a regular triangulation by imposing an edge length of h=215μm, and finally we mapped the model parameters from the original mesh to the smooth refined one using a nearest neighbour projection. Details on the generated computational meshes can be found in the online supplement. The model was discretised in space with linear finite elements; the non-linear term describing the ionic current was treated with a splitting technique, Pathmanathan et al. (2012) and Whiteley (2006). The cell model was discretised in time with a forward-Euler scheme and a time step of $dt_{\mathrm{ODE}}=5\;\mu s,$ while the diffusive parabolic PDE was implemented with a Crank–Nicholson scheme and a time step of $dt_{\mathrm{PDE}}=50\;\mu s$. We performed numerical simulations with Cardiac Arrhythmias Package (CARP), an electrophysiology solver suitable for high-performance computing.

### 2.9 External stimulus

To initiate the numerical simulations, we apply an external stimulus of intensity $I_{\mathrm{app}}=4\;{m\;s}^{-1}$ (this units reflects the dimensions of the model equations introduced in Mitchell, Schaeffer, 2003, Corrado, Niederer, 2016) and duration $t_{\mathrm{stim}}=\;0.6$ ms, on a circular region of radius $R_{\mathrm{stim}}=1\;\mathrm{cm}$. The activation region was defined as a 1cm disk to reflect the uncertainty in the activation location. This current approximates the activation of the LA from the wave propagating from the pacing site (CS or HRA) in the clinical procedure. As activation times were not recorded across the entire LA we cannot determine the location of the first point of activation in the LA directly from the clinical data. For both CS and HRA stimulations the stimulus was applied at the first point of activation on the LA endocardium. As these two electrodes are not in direct contact with the left atrial endocardium the activation site was estimated. To determine the first activation site in the LA and hence the location of the stimulus in the model we apply the following procedure. First, on the computational mesh produced by the electroanatomical mapping system, we extrapolated the local CV at the largest coupling interval ($s_{2}=343$) over the whole domain using the nearest-neighbours criterion, as described above for the model parameters. We then use the CV values to estimate the LATs over the entire LA using an eikonal model solved with a graph-based method (Wallman, Smith, Rodriguez, 2012, Corrado, Zemzemi, 2018). Second, to identify the point in the mesh where the activation starts, we loop over each point in the mesh predicting the LAT pattern if the candidate point was the first point to be activated. This gives an LAT map corresponding to each point in the mesh. Third, to exclude any systematic error produced by the time required by the electrical stimulus to reach the simulated stimulation position from the real stimulation position, we shifted the computed LATs with an offset equal to the mean difference between the measured and the computed LATs. Forth, we then evaluated for each point the mean absolute error between the measured LATs and the times computed with the eikonal model. Finally, we identified the centre of the external stimulus as the point that produced the minimum error. Further details of this procedure can be found in the online supplement.

### 2.10 Validating the atrium models

We validated the patient specific models generated for each clinical case by comparing the LATs measured during the clinical procedure and the LATs obtained from the numerical simulations. We first tested if the model was able to reproduce the data used to build the patient specific model; we thus simulated the LATs obtained when the stimulus is applied on the CS and then we compared the numerical solution with the data recorded during the clinical procedure. Next, we tested the predictive performance of the personalised model by reproducing the HRA pacing activation time of measurements that were not used to constrain the parameters. None of the patients considered in this study received an MRI, so we do not have anatomical information to describe either the location of the heart relative to the torso, or the location and the shape of the right atrium relative to the left atrium. Thus, we have not simulated the right atrium, and we are unable to perform meaningful simulations of P waves to inform the validation process. As before, we shifted the computed LATs with an offset equal to the mean difference between the measured and the computed LATs.

For each coupling interval *s*_2_, we evaluated the correlation coefficients between the measured LATs and the LATs at the electrodes obtained from numerical simulations; some simulations presented regions that were not activated as a consequence of local refractoriness; this acted as a functional block and prevented the activation wave propagating further. In some cases, this resulted in downstream electrodes not being stimulated and hence not activating. The proportion of non-activating electrodes in model simulations are reported in the function block index. We took into account this type of error, defining the error on the functional block as the ratio between the total number of functional block occurrences at the electrodes in the numerical simulations, and the total number of available LATs measurements at the electrodes. We evaluated the following quantities with the measured and computed LATs on all the electrodes and for all the considered values of *s*_2_:

• *The regression line*, y=mx+q. As the coefficient m is close to 1 and the coefficient *q* is close to 0, as the model better represents the experiments.
• *The Pearson correlation coefficient, r*. As this coefficient is close to 1 as the simulations are a better representation of the patient.
• *The “covariance slender ratio”*, sl. This coefficient corresponds to the ratio between the two principal components of the covariance matrix. As this coefficient is close to 0, as the model is a better representation of the data. The covariance between measured and computed LATs is graphically represented with the red ellipses depicted on Figs. 5 and 6.
• *The functional block error*, fblock error. This coefficient is a measure of the electrodes in the computational model where an activation was not measured. This error is expressed as a percentage and it is defined as follows:
  $$\text{fblock}\;\text{error}=100\cdot \frac{\sum_{i_{s_{2}}}Np_{i_{s_{2}}}^{\mathrm{eval},\text{block}}}{\sum_{i_{s_{2}}}Np_{i_{s_{2}}}^{\mathrm{meas}}}$$
  where, fixed the coupling interval *s*_2_, $Np_{i_{s_{2}}}^{\mathrm{meas}}$ is the number of electrodes where an LATs was measured and $Np_{i_{s_{2}}}^{\mathrm{eval},\text{block}}$ is the number of electrodes where no activation was measured in the computed solution.

The distributions of the absolute and relative errors between the simulated and the measured LATs, together with their mean and standard deviations can be found in the online supplement.

## 3 Results

**Fig. 2:**
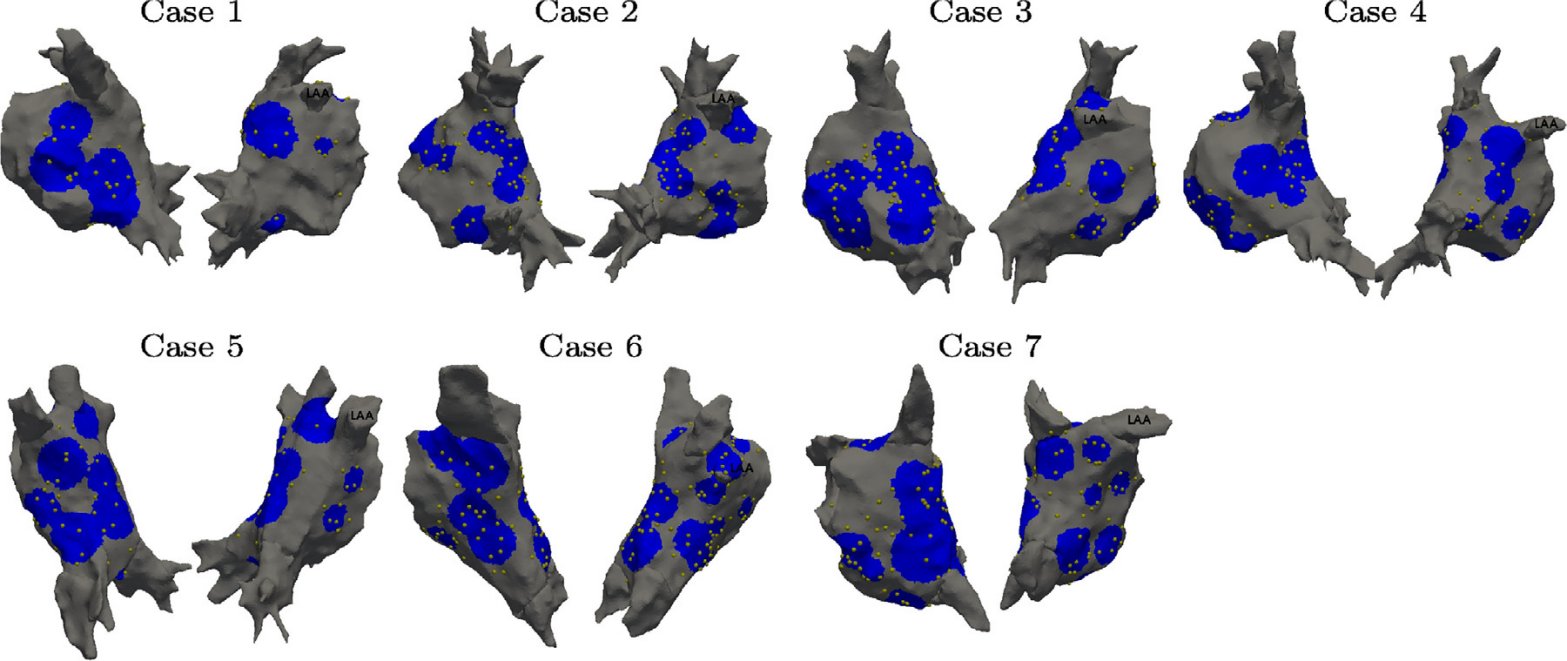
Anatomies for clinical cases 1–7. The gold spheres represent the position of the electrodes. The blue region represents the atrium surface covered by measurements. (For interpretation of the references to colour in this figure legend, the reader is referred to the web version of this article.)

In this section, we present the validation of the locally personalised models on the 7 clinical cases characterised by the anatomies shown in Fig. 2. On the same figure, we marked with gold spheres the recording electrodes presenting at least one EGM trace. For each site the catheter was manoeuvred, we evaluated a circular region centred at the barycentre of the catheter electrodes and with radius equal to the mean distance between the electrodes and the electrodes barycentre. We considered the union of these circular regions as the atrial surface covered by the catheter during the procedure and marked in blue on Fig. 2.

**Fig. 3:**
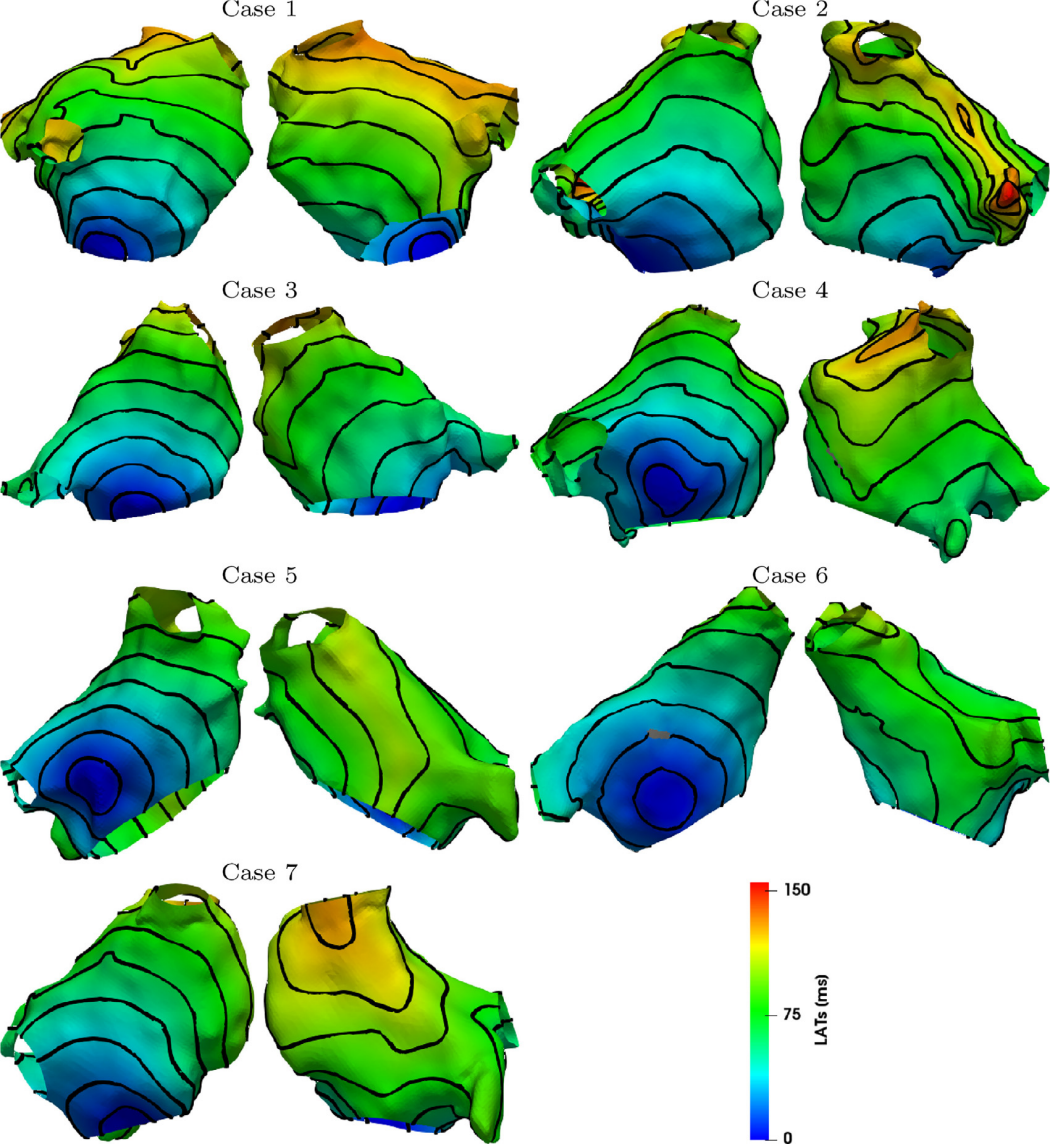
LATs evaluated by numerical simulations when $s_{2}=343\;\mathrm{ms}$ and stimulus is applied in the CS. Isochrones are equally spaced with 10 ms intervals.

**Fig. 4:**
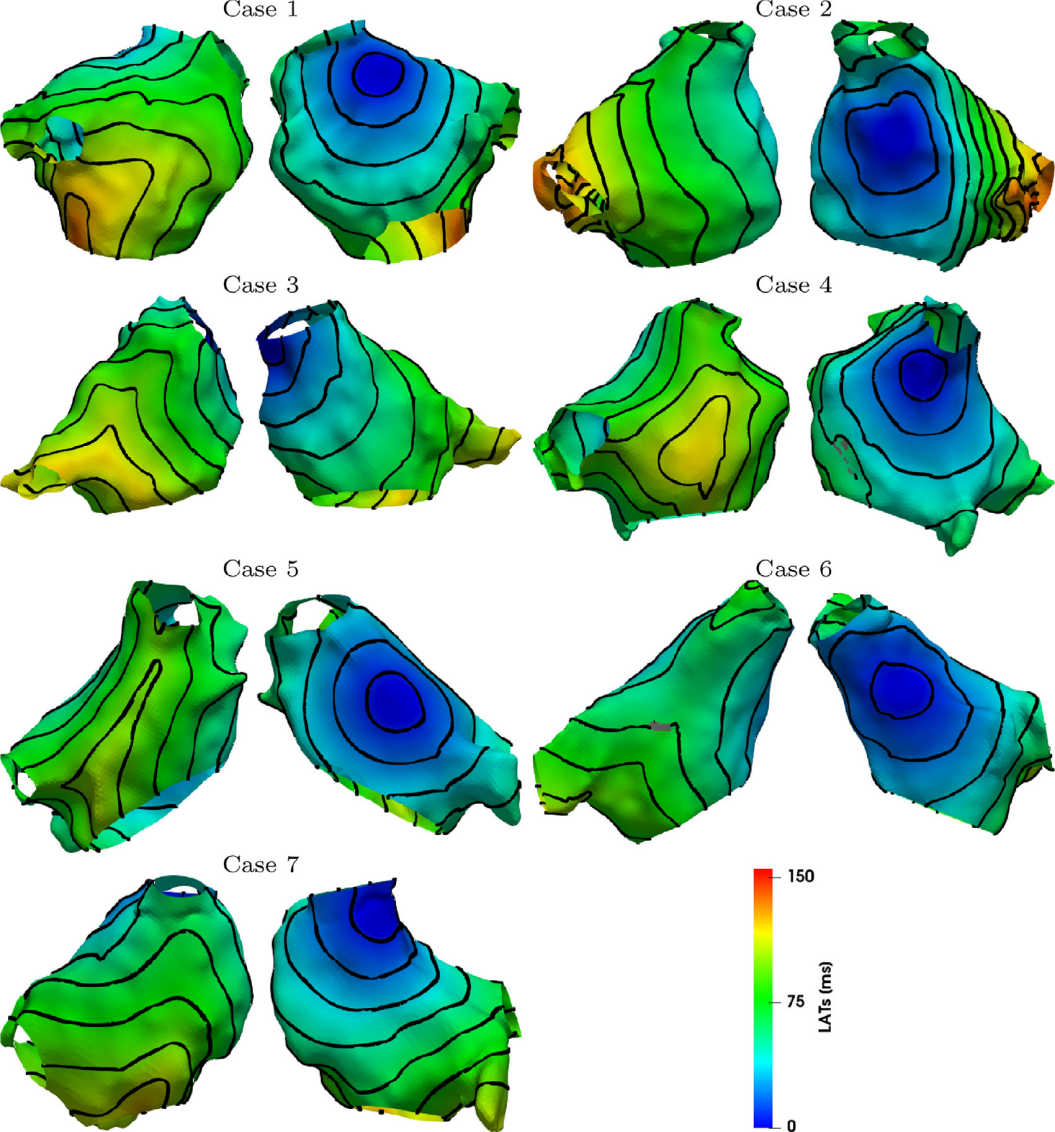
LATs evaluated by numerical simulations when $s_{2}=343\;\mathrm{ms}$ and stimulus is applied at HRA. Isochrones are equally spaced with 10 ms intervals.

We apply the validation procedure described in Section 2.10 for each of the 7 clinical cases; Figs. 3 and 4 show an example of LATs obtained from numerical simulations for $s_{2}=343$ ms and the external stimulus applied in the CS or in the HRA. On the same figure, we depicted 10 ms equally spaced isochrones.

**Fig. 5:**
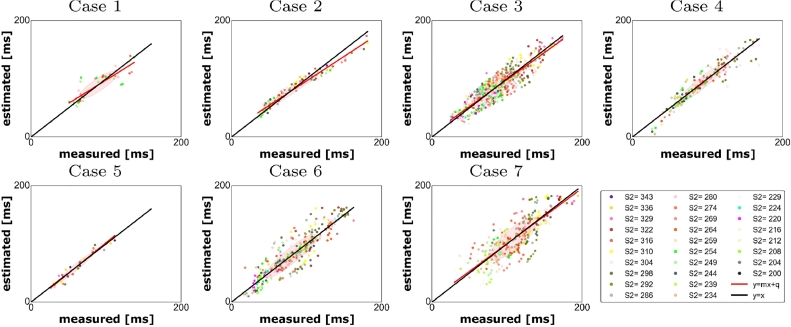
Measured vs estimated activation times for the personalised model (CS). Each point represents a measured vs computed LATs at each electrode and for each *s*_2_; each colour represents the measurements taken at the electrodes for a fixed *s*_2_ value.

**Fig. 6:**
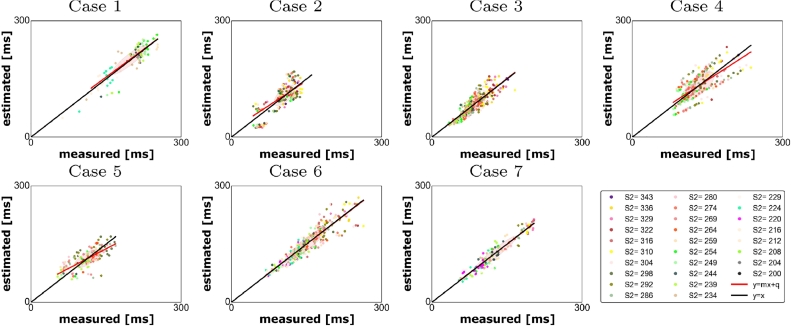
Measured vs estimated activation times for the personalised model (HRA). Each point represents a measured vs computed LATs at each electrode and for each *s*_2_; each colour represents the measurements taken at the electrodes for a fixed *s*_2_ value.

We first tested how the model reproduces the CS pacing LATs that we used to constrain the model parameters. In this case, the correlation between measured and simulated LATs (Fig. 5) ranged between 0.81 and 0.98. The model did not predict 2.59% of the activations (case 3) as reported in Table 2.

**Table 2:** Left: indicators used to estimate the accuracy in reproducing the experiments when a model with locally personalised electrophysiology is adopted (CS); right: indicators used to estimate the accuracy in reproducing the experiments with the same model and pacing on HRA.

Case	(*q, m*)	*r*	sl	fblock error (%)	Case	(*q, m*)	*r*	sl	fblock error (%)
1	(17.52, 0.8)	0.87	0.07	0.00	1	(11.23, 0.96)	0.90	0.05	41.18
2	(11.88, 0.84)	0.98	0.01	0.0	2	(17.88, 0.85)	0.65	0.19	0.00
3	(5.93, 0.93)	0.91	0.05	2.59	3	(−1.23, 1.02)	0.86	0.07	0.00
4	(0.12, 1.0)	0.94	0.03	1.1	4	(21.36, 0.84)	0.73	0.15	0.00
5	(−3.12, 1.05)	0.97	0.02	0.00	5	(36.44, 0.68)	0.73	0.15	9.73
6	(−0.06, 1.0)	0.91	0.05	1.12	6	(1.74, 0.99)	0.92	0.04	1.48
7	(5.24, 0.95)	0.81	0.10	0.65	7	(1.43,0.99)	0.96	0.02	0.0

Next, we validated the model against the LATs we measured during HRA pacing by simulating the HRA pacing protocol in the model. Since this set of data was not used to constrain the model parameters, this test allows us to assess the predictive performance of the personalised models. In this case, the correlation between measured and simulated LATs (Fig. 6) ranged between 0.65 and 0.96. In one case (case 1, Table 2), the model did not predict 41.18% of the activations. In the proximity of the external stimulus, we measured a region with a large ERP (ERP  >  300 ms): as we interpolated the parameters with a nearest neighbour criterion, we obtained an overestimation of the size of the region with this large ERP value, that superimposed on most of the region were stimulus is applied causing a large area not to be activated. This large error is likely due to insufficient data and small coverage, especially on the anterior wall. Hence, at least 50 data points covering most of the regions of LA are required for making a model. For each case, the quantities introduced in Section 2.10 are summarised in Table 2 for the models with pacing on CS (left) and on HRA (right).

## 4 Discussion

In this paper, we validated a method to generate locally personalised computational models of the left atrium from clinical measurements. When planar propagation was considered, we obtained a correlation between the predicted and the measured atrial function ranging between 0.65 and 0.96 (median 0.86) with a level of uncertainty on the predicted activation ranging between the 2% and 19% (median 7%) of the data variability. The maximum value of the absolute error on the local activation times was 60 ms when the stimulus was applied in the CS and 80 ms when the stimulus was applied in the HRA (online supplement). The standard deviation of the absolute error in the model ranged from 12.84 ms to 25.79 ms in the model fitting and 12.99 ms to 27.13 ms in the model validation (online supplement). Further, these values are comparable with those reported in Sermesant et al. (2012) for ventricles. These numbers need to be placed within the context of the size of the bipolar electrode (7 mm) and the mean conduction velocity ( ∼ 1 m/s) so the activation time across the electrode itself is 7ms. This study makes four main contributions. First, we have proposed a data assimilation technique for combining electroanatomical mapping data across the atrium into a single common physically constrained framework. Second, we have extended our clinical parameter estimation method using the mMS model and determined the database parameter span based on leading order approximations of physiological readouts. Third, we used an eikonal method to identify the first point of activation from sparse LAT clinical recordings. Forth, we have shown that it is possible to generate and validate patient specific models of atrial electrophysiology that capture frequency response and electrophysiological heterogeneity using electroanatomical mapping recordings.

*Data distributions.* Globally, when we stimulated in the CS, we measured conduction velocities with a mean of 88 cm/s and a standard deviation of 35 cm/s; when we stimulated in the HRA, we measured conduction velocities with a mean of 112 cm/s and a standard deviation of 49cm/s.

For each coupling interval *s*_2_, the conduction velocities obtained stimulating in the HRA presented mean values  ∼ 20 cm/s faster than those obtained when stimulating in the CS.

The effective refractory periods spanned between 200 and 322 ms, with a mean value of 239 ms and a standard deviation of 30 ms (CS), and between 212 and 322 with a mean value of 246 ms and a standard deviation of 24 ms (HRA).

When we stimulated in the CS, on the Anterior wall we measured conduction velocities ranging between 50 and 133 cm/s, with a mean standard deviation of 36 cm/s that varies with *s*_2_ in the range 3–59 cm/s; on the Posterior wall we measured conduction velocities ranging between 76 and 98 cm/s, with a mean standard deviation of 16 cm/s that varies with *s*_2_ in the range 5–29 cm/s. On the left atrial Roof, we measured conduction velocities ranging between 49 and 94 cm/s, with a mean standard deviation of 33 cm/s that varies with *s*_2_ in the range 8–56 cm/s. Finally, on the left atrial Floor, we measured conduction velocities ranging between 64 and 115 cm/s, with a mean standard deviation of 22 cm/s that varies with *s*_2_ in the range 9–37 cm/s.

On each of the regions considered here, we measured a minimum value of the ERP of 200 ms. For the Anterior wall, Posterior wall, atrial Roof and atrial Floor we measured maximum ERP values of 280, 316, 316 and 304 ms, respectively, mean values of 230 252 228 and 236 ms and standard deviations of 20, 30 25 and 31 ms, respectively.

When we stimulated in the HRA, on the Anterior wall we measured conduction velocities ranging between 59 and 118 cm/s, with a mean standard deviation of 34 cm/s that varies with *s*_2_ in the range 3–88 cm/s; on the Posterior wall we measured conduction velocities ranging between 82 and 126 cm/s, with a mean standard deviation of 31 cm/s that varies with *s*_2_ in the range 6–52 cm/s. On the left atrial Roof, we measured conduction velocities ranging between 54 and 143 cm/s, with a mean standard deviation of 26 cm/s that varies with *s*_2_ in the range 2–47 cm/s. Finally, on the left atrial Floor, we measured conduction velocities ranging between 79 and 133 cm/s, with a mean standard deviation of 37 cm/s that varies with *s*_2_ in the range 15–50 cm/s.

For the Anterior wall, Posterior wall, atrial Roof and atrial Floor, we measured minimum values of the ERP of 212 220 212 and 208 ms respectively, maximum values of 298 274 304 286, respectively, mean values of 249 244 257 and 241 ms and standard deviations of 27, 21 31 and 22 ms, respectively. The median values were 244 234 257 and 234 ms, respectively.

*Data Assimilation.* Inferring heterogeneous parameter values from sparse data is challenging. Previous methods based on Kalman filtering (Corrado et al., 2015) require solving one or more direct problems and then sequentially modify the parameter values proportionally to the discrepancy between the measurements and the model. This procedure requires the subdivision of the myocardium into a set of regions characterised by uniform parameters, limiting the resolution on the degree of heterogeneity. At each sampling iteration, the algorithm implementing the Kalman filter inverts a covariance matrix that is full and with a size equal to the number of parameters to estimate; the computational demands of this process, in particular as far as the solution of the direct problems is concerned, hampers the application of this technique to clinical applications. We have developed a computationally efficient and robust method that relies on a large pre computed database of results (Corrado et al., 2016a) to ensure an optimal parameter set can be found at each location. This method allows local tissue properties to be inferred, independent of the whole organ model and allows parameters to be constrained on a clinical time scale.

*Cell model.* We described the electrical activity of the cell membrane with the mMS cell model (Corrado and Niederer, 2016); this model was proven to be stable to pacemaker behaviour (Corrado and Niederer, 2016), and to furnish spiral waves characterising tachycardia and AF, Corrado et al. (2016b) equivalent to those obtained adopting the MS model (Corrado et al., 2016a). The stability to pacemaker behaviour reduces the time required to apply the whole process since tests on the stability are no longer required. Using the mMS model personalisation allows cellular readouts (CV, ERP) to be analytically estimated using leading order approximations. Even though inaccurate, the leading order expressions approximates the value of the cellular readouts for a chosen parameter set and their sensitivities with respect to the parameters. As the CV and APD are physiological properties with known bounds it is possible to use this information to constrain the distribution of the parameter estimation data set and to adopt an increment on the parameter values that produces a significant variation on measurements. This ensures that the database covers a physiologically plausible space and that parameters in the database are distributed evenly over that space.

*Stimulus location.* To perform numerical simulations of electrical activation the location of the electrical stimulus must be determined. When the stimulus is applied at the CS this point is still on the left atrium, however, the location of the catheter along the CS is not known; when the stimulus is applied on the HRA one has to identify the region where the activation front propagates from the right atrium and then first enters the left atrium. The coarse resolution of the measurements used in this study (Williams et al., 2017a), the partial coverage of the atrial surface and the irregular geometry of the atrium anatomies prevent using the method proposed in Fitzgerald et al. (2005), where stimulus locations are determined evaluating the divergence of the conduction velocity field. We determined the stimulus location by approximating the depolarisation front propagation with an eikonal model and comparing the predicted LATs with the measured ones considering each point of the electroanatomical mesh as onset. To obtain an estimate of the heterogeneous CV distribution across the whole atrium, we interpolated the values of CV with the same nearest neighbour criterion adopted for the parameters. Differently from Konukoglu et al. (2011), in this study we did not model the uncertainty on the parameters on the region where measurements were unavailable, while we adopted an efficient graph-based algorithm (Wallman, Smith, Rodriguez, 2012, Corrado, Zemzemi, 2018) for solving the eikonal model; this enabled testing each point of the tetrahedral mesh generated by the electroanatomical mapping as onset candidate and then choosing the candidate yielding the minimum least square error evaluated at the electrode positions.

## 5 Limitations

The challenge of making computational models from clinical data are significant. Patients are complex and require a lot of data to constrain their individual physiology. At the same time recoding within the clinical environment places clear constraints on the data collected. Here we discuss some of the challenges that we have identified in the collection and analysis of the data in our model that place clear caveats on the model and also provide practical considerations for future data collection and model development.

Clinical measurements are inherently noisy. Even using a PentaRay catheter, often some electrodes are unable to capture any signal thus reducing the spatial resolution of the method. Parameters are interpolated on regions where no recordings are available via a nearest neighbour criteria; when the spatial resolution of clinical measurements is too coarse, this method could present an overestimation of regions characterised by a localised abrupt variation of the local electrophysiology properties, such as scar or fibrotic regions; a possible solution to reduce the uncertainty on the boundaries of these regions could be represented by enriching the spatial distribution of the catheter data with data obtained by an imaging technique, such as MRI or CT scan. However this data was not available on the 7 cases we have considered here.

Differently from the cases treated in Corrado et al. (2016a), in this paper the electrical stimulus is applied with a remote catheter, placed either in the CS or in the HRA. This procedure presents several issues: first, since we adopt a bipolar configuration of the measurement electrodes, we can no longer be sure that the activation wave is propagating in an orthogonal direction from the measurement catheter. Second, the probability that the tissue is activated by the catheter stimulus rather than the normal sinus rhythm decreases when the distance between the pacing and the recording sites increases. This may cause a discrepancy between the applied *s*_2_ and the locally experienced *s*_2_ duration. However, as we applied the stimulus in a continuous drive train it is unlikely that we experienced significant sinus rhythm artefacts. Third, when the distance between the pacing and the recording electrodes increases, the restitution properties at the recording electrodes are characterised by the local interval between two subsequent activations, *A*_1_*A*_2_, rather than the inter-pacing interval *s*_1_*s*_2_. As a consequence, the inter-pacing interval at the electrode location depends on the way the stimulus propagates across the tissue. For shorter values of *s*_2_, the correlation between two subsequent stimuli *s*_1_*s*_2_ and two subsequent activations, *A*_1_*A*_2_ is no longer linear, thus introducing an additional uncertainty. Fifth, when the stimulus is applied at HRA, it is possible that the left atrium is activated at more than one location leading to a depolarisation triggered by two propagation fronts coming from two different directions. The absence of local minima in the cost function is consistent with no second stimulus being present in the areas covered by our measurements. However, we are unable to rule out the presence of second activation sites that have a limited impact on the proposed cost function. Sixth: we have not included conduction anisotropy in the model. Locally we did not see any compelling evidence for tissue anisotropy in the activation patterns. However, this is potentially due to the pacing protocol. When the stimulus is applied on a remote electrode, the measuring tissue anisotropy requires recording two activation waves that propagate over the same piece of tissue, ideally from two orthogonal directions that align perfectly with the fibre and cross fibre direction. The pacing protocol we adopted in this work was not optimised to generate anisotropy measurements as pacing sites were chosen as two routine clinical pacing locations. In future studies, pacing from the centre of a PentaRay catheter and measuring a local elliptical activation pattern is one option, but requires enough PentaRay splines to be in contact to measure activation times spread around the stimulating electrode, which is not guaranteed and may lead to bias in the measurement locations. The atrium has a complex transmural fibre orientation, while electro-anatomical mapping records data on the endocardial surface only. Thus, endocardial recordings are able to measure anisotropy in regions where transmural fibres align to endocardial fibres; regions where transmural fibres are orthogonal to endocardial fibres may appear as isotropic. As part of the model creation process, we have performed a validation study where we compare our model predictions to clinical data not used to constrain the model. Despite the noise in the measurements, the model limitations and the coverage and resolution of the measurement, we obtain a strong consistency (see Figs. 5 and 6 and Table 2) between the predictions and measurements. Thus, while anisotropy may be important in specific conditions it appears less important for predicting endocardial activation patterns during pacing.

Seventh: the model was fitted to a standard *s*_1_*s*_2_ pacing protocol (Murgatroyd, 2002, Narayan, Kazi, Krummen, Rappel, 2008) with *s*_2_ spanning 343 ms down to 200 ms, approaching activation intervals close to the cycle lengths seen in some AF mapping studies (Haïssaguerre et al., 2007). The activation pattern in AF is complex. Depending on the driver of AF, (mother rotor, ectopic beats or multiple wavelets (Schricker et al., 2014)), the activation rates locally may be more or less regular. The protocol we have chosen captures the dynamic changes in pacing rate and may not characterise the electrical dynamics at faster stable activation rates. However, the cell model that we use captures the characteristic monotonic decreases in ERP and CV as pacing rate accelerates so that the model provides a physiologically plausible estimate of the electrophysiological dynamics at faster rates that may be seen in AF.

## 6 Conclusions

In this paper, we presented a pipeline to generate locally personalised computational model of the left atrium from multi-polar catheter measurements, obtained during a clinical procedure. We then applied the method to 7 data sets recorded from paroxysmal AF patients undergoing pulmonary vein isolation. The method we presented is able to predict personalised atrial activation times using models that capture an individuals heterogeneous electrophysiology and thus paves the way to the study of atrial fibrillation and computer guided radio-frequency ablation procedure.

## Appendix A Supplementary materials

Supplementary Data S1 Supplementary Raw Research Data. This is open data under the CC BY license http://creativecommons.org/licenses/by/4.0/ Supplementary Data S1
