# Supplementary material for: A work flow to build and validate patient specific left atrium electrophysiology models from catheter measurements
Source: Med Image Anal. 2018 Jul;47:153–63. doi: 10.1016/j.media.2018.04.005 (PMC5998385; doi:10.1016/j.media.2018.04.005)
Supplement: Supplementary Data S1 — Supplementary Raw Research Data. This is open data under the CC BY license http://creativecommons.org/licenses/by/4.0/ [file mmc1.pdf]

Online Supplement: A Work Flow to Build and Validate Patient Specific Left Atrium Electrophysiology Models from Catheter Measurements

C. Corrado, S. Williams, R. Karim, G. Plank, M. O'Neill, S. Niederer

1 Measurements

Table 1 summarises the number of recordings available for each clinical case when the heart is paced from the coronary sinus (CS) or the high right atrium (HRA). These quantities correspond to the total number of electrodes presenting at least one electrogram (EGM) recording for any of the values of the premature stimulus s_2. On the same table we also report the total number of sites that the PentaRay catheter was manoeuvred to during the study. A total of 28 s_2 coupling intervals were tested for each electrode site and for each pacing location.

Case	1	2	3	4	5	6	7
CS meas	49	68	95	60	36	100	95
HRA meas	42	91	80	64	51	98	86
number of sites	12	13	14	12	12	15	16

Table 1: Number of electrodes presenting at least one EGM trace when stimulus is applied in the proximity of CS (first row) or in the HRA (second row); number of sites the PentaRay catheter was manoeuvred during the procedure (third row).

Figure 1 shows the 7 anatomies of the left atria obtained from the electro anatomical mapping system (Ensite Velocity St Jude). The Ensite Velocity anatomy consists of a triangulation of the atria surface and provides a mathematical description of the anatomy for mapping measured properties onto spatial coordinates. On the same figure, we marked with gold spheres the recording electrodes presenting at least one EGM trace.

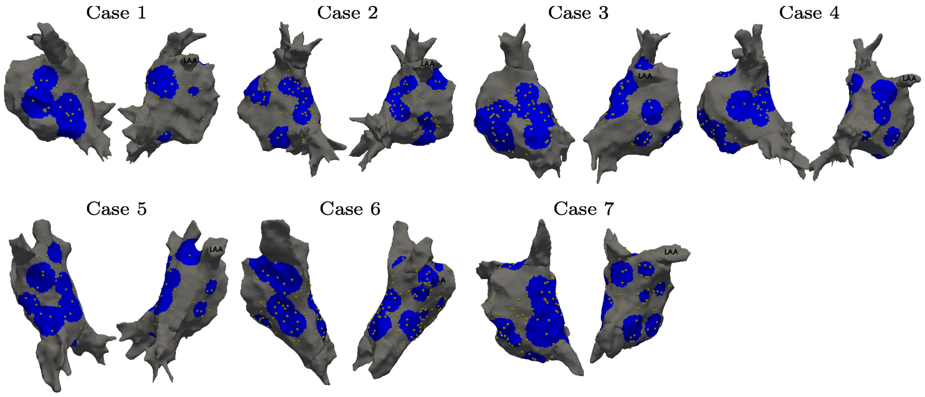

Figure 1: Anatomies for clinical cases 1-7. Gold spheres represent the position of the recording electrodes that presented at least one EGM trace. For each site the catheter was manoeuvred, a circular region centred at the barycentre of the catheter electrodes and with radius equal to the mean distance between the electrodes and the electrodes barycentre, was evaluated. The union of these regions was considered as the atrial region covered by the PentaRay catheter and marked in blue.

For each site the PentaRay catheter was manoeuvred, we evaluated a circular region centred at the barycentre of the catheter electrodes and with radius equal to the mean distance between the electrodes and the electrodes barycentre. We considered the union of these circular regions as the atrial surface covered by the catheter during the procedure and marked in blue on Figure 1; in Table 2 we also reported the corresponding percentage of atrial surface covered by measurements.

Case	1	2	3	4	5	6	7
Percentage covered(%)	10.3	8.9	23.2	13.6	10.4	23.3	18.7

Table 2: Percentage of the left atrium surface covered by measurements.

2 Measured Conduction Velocity Distribution

Figure 2 shows the distribution of calculated CV for all the 7 clinical cases and for all the s_2 coupling interval considered. On the left panel we plotted the distribution of CV obtained when the external stimulus is applied at CS; on the central panel we plotted the distribution of CV obtained when the external stimulus is applied on the HRA. On the right panel we plotted the distribution of CV obtained from both data sets.

The measured distributions are consistent with those reported in [7, 4, 8] and were used to select a value of CV=200 cm/s as a threshold for identifying outliers.

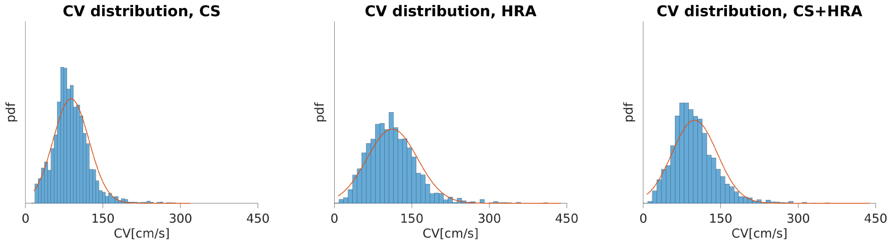

Figure 2: CV distribution for an external stimulus applied in the proximity of CS (left), and on the HRA (centre); overall CV distribution (right).

Table 3 summarizes the mean and the standard deviations of the CV distributions.

	CS	HRA	CS+HRA
μ [cm/s]	88.13	112.1	99.51
σ [cm/s]	34.66	48.84	43.66

Table 3: Mean and standard deviation for the CV distributions shown in Figure 2

3 Re-parametrisation of the data set

For the ionic model [3] it is possible to define leading order approximations of APD and CV restitutions curves.

In this work, a parameter grid was built τ_{in}, τ_{open} and on CV_{max}, APD_{max} h_{min}, defined as follows:

$$h_{\text{min}} = \left(1 + \frac{\tau_{\text{out}}}{4\tau_{\text{in}}}(1 - v_{\text{gate}})^2\right)^{-1}$$

$$\text{APD}_{\text{max}} = \tau_{\text{close}}\left(\frac{1}{h_{\text{min}}}\right)$$

$$\text{CV}_{\text{max}} = \frac{1 - 2v_{\text{gate}}}{2}\sqrt{\frac{2D}{\tau_{\text{in}}}}$$

Once a set of parameters is defined, we can obtain the original parameters characterising the mMS model from expressions (1):

$$
\begin{aligned}
&\left(\mathrm{CV}_{\max}, \tau_{\mathrm{in}}, h_{\min}, \tau_{\mathrm{open}}, \mathrm{APD}_{\max}\right) \rightarrow \left(D, \tau_{\mathrm{in}}, \tau_{\mathrm{out}}, \tau_{\mathrm{open}}, \tau_{\mathrm{close}}\right) \\
D &= \frac{\tau_{\mathrm{in}}}{2}\left(\frac{2\mathrm{CV}_{\max}}{1-2v_{\mathrm{gate}}}\right)^2 \\
\tau_{\mathrm{in}} &= \tau_{\mathrm{in}} \\
\tau_{\mathrm{out}} &= \frac{4\left(1-h_{\min}\right)\tau_{\mathrm{in}}}{h_{\min}\left(1-v_{\mathrm{gate}}\right)^2} \\
\tau_{\mathrm{open}} &= \tau_{\mathrm{open}} \\
\tau_{\mathrm{close}} &= -\frac{\mathrm{APD}_{\max}}{\log\left(h_{\min}\right)}
\end{aligned} \tag{1}
$$

The parameters $h_{\min}$ and the leading order of $\mathrm{APD}_{\max}$ are defined in [3]; in section 3.1 we derive a leading order approximation for CV as a function of the diastolic interval (DI) and then the expression for $\mathrm{CV}_{\max}$ as the limit for DI $\rightarrow \infty$.

3.1 Asymptotic derivation of the CV restitution curve

We obtain a leading order expression for the CV restitution on a 1D homogeneous filament characterised by a homogeneous diffusivity coefficient σ and the ionic model defined in [3]:

$$
\begin{aligned}
\partial_t v_{\mathrm{m}} &= D\partial_{xx} v_{\mathrm{m}} + I_{\mathrm{ion}}\left(v_{\mathrm{m}}, h\right)\left(h, v_{\mathrm{m}}\right) \\
I_{\mathrm{ion}}\left(v_{\mathrm{m}}, h\right)\left(h, v_{\mathrm{m}}\right) &= \frac{h v_{\mathrm{m}}}{\tau_{\mathrm{in}}}\left(v_{\mathrm{m}}^{+}\left(h\right) - v_{\mathrm{m}}\right)\left(v_{\mathrm{m}} - v_{\mathrm{m}}^{-}\left(h\right)\right)
\end{aligned} \tag{2}
$$

where $v_{\mathrm{m}}^{\pm}$ define the expressions of the left and right null-clines,[3]. When the depolarisation front propagates, it is possible to approximate $h \simeq \mathit{const}$, since usually $\tau_{\mathrm{in}} \ll \tau_{\mathrm{out}} \ll \tau_{\mathrm{open}}, \tau_{\mathrm{close}}$. We then introduce the coordinate $z = x - \mathrm{CV} \cdot t$, centred on the propagation front and moving with the front propagation velocity and substitute this definition into equation (2), leading to the following second order ODE in $U(z)$:

$$
\begin{aligned}
&DU'' + \mathrm{CV}\, U' + I_{\mathrm{ion}}\left(v_{\mathrm{m}}, h\right)\left(U\right) = 0 \\
&\lim_{z\rightarrow -\infty} U(z) = v_{\mathrm{m}}^{+} \qquad \lim_{z\rightarrow \infty} U(z) = 0 \\
&\lim_{z\rightarrow \pm\infty} U'(z) = 0
\end{aligned} \tag{3}
$$

where primes denote derivation w.r.t. z. Equation (3) also satisfies the following condition, [6]:

$$
U' = c_0 U\left(U - v_{\mathrm{m}}^{+}\right) \tag{4}
$$

where c_0 is a constant to determine. Substituting (4) in (3) and after some calculus we obtain:

$$c_0 = \sqrt{\frac{h}{2\tau_{\text{in}} D}}$$

$$\text{CV} = \sqrt{h\frac{2D}{\tau_{\text{in}}}}\left(\frac{1}{2}v_{\text{m}}^{+} - v_{\text{m}}^{-}\right)$$

Finally, we evaluate $\text{CV}_{\max}$ as the limit of CV when $\text{DI} \to \infty$:

$$\lim_{\text{DI}\to\infty} \text{CV} = \text{CV}_{\max} = \frac{1 - 2v_{\text{gate}}}{2}\sqrt{\frac{2D}{\tau_{\text{in}}}}$$

4 Fitting of CV restitution

Figure 3 shows the local CV restitutions measured and fitted at 20 evenly sampled electrodes and for each clinical case. The blue lines represent the CV restitutions we obtained from clinical measurements; the black lines represent the local restitutions we obtained from the fitting algorithm described in [1, 2] when no space regularisation is applied and the red lines represent the restitutions we obtained from the fitting when we applied the space regularisation.

5 Estimated parameters

Figures 4 - 8 show the spatial distribution of the conductivity and of the ionic parameters, obtained after the parameter interpolation on the atrial surface; to improve the readability we unfolded the atrial surface [5] on a disc and we removed the left atrial appendage (LAA). On the same figure, we marked the recording electrodes with gold spheres.

6 Computational meshes

Table 4 summarizes the number of points, elements and the characteristic mesh sizes for each of the 7 mesh we generated. For each case, Table 5 summarizes the number of points in the Velocity mesh that was generated by the electroanatomical mapping system and to reconstruct the atrial anatomy.

7 Stimulus location

Figure 9 shows the rescaled error distribution on pacing site location for the stimulus applied in the proximity of CS, while figure 10 shows the same quantity for the stimulus applied at HRA. Gold spheres represent the locations that best fit data. The region of lowest error in Figure 9 is in agreement with the anatomical position of the respective stimulation electrode.

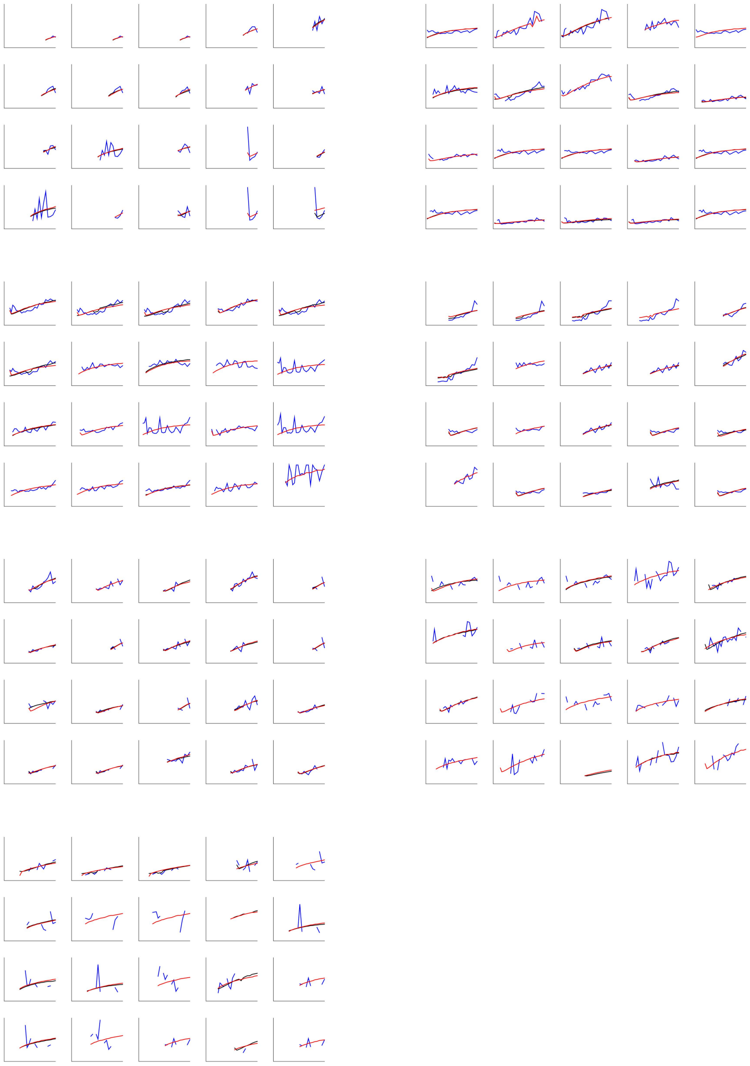

Figure 3: Local CV restitutions at 20 evenly sampled electrodes and for each clinical case. Blue lines represent the measured restitutions; black lines represent the fitted CV restitutions obtained by directly applying the fitting algorithm. Red lines represent the fitted restitutions when also a space regularisation is applied. Values of s_2 (x axis) range between 200 ms and 343 ms; values of CV (y axis) range between 0 and 200 cm/s.

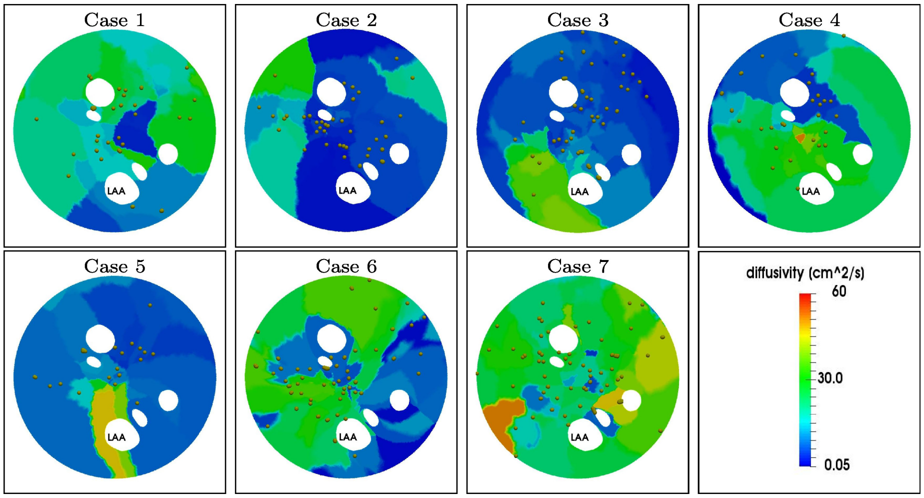

Figure 4: Distribution of the diffusion on the atrial surface for each of the 7 cases

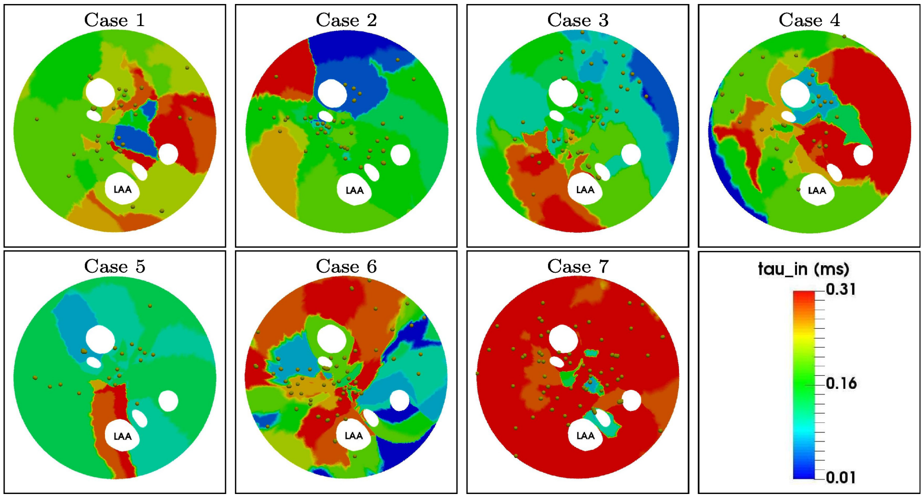

Figure 5: Distribution of $\tau_{\rm in}$ on the atrial surface for each of the 7 cases

Case	nb of vertices	nb of triangles	min edge size μm	avg edge size μm	max edge size μm
1	952937	1903426	56.27	177.58	784.82
2	1087668	2172758	63.87	177.56	678.27
3	997031	1991426	29.62	177.61	662.85
4	1113896	2224887	39.50	177.62	844.81
5	1052004	2101151	37.89	177.65	689.21
6	1047446	2092374	31.55	177.57	668.67
7	1014700	2027005	32.26	177.60	756.38

Table 4: Mesh properties for each of the 7 anatomies.

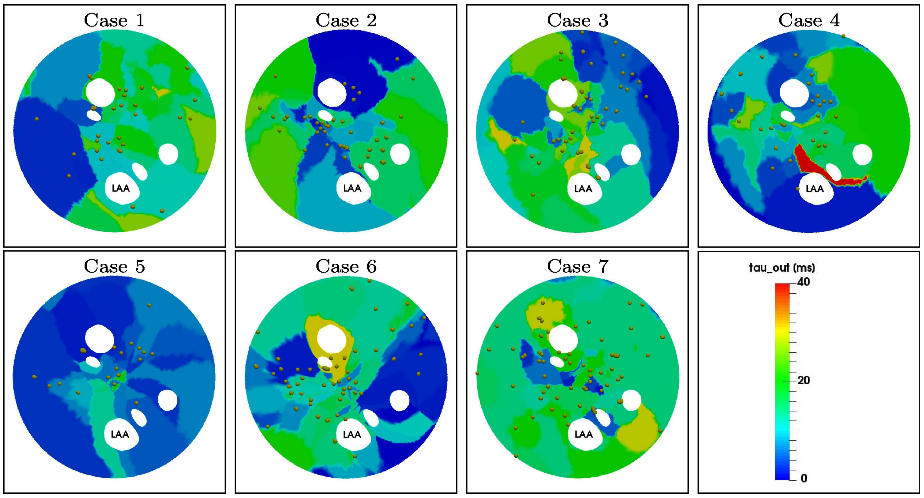

Figure 6: Distribution of τ_{out} on the atrial surface for each of the 7 cases

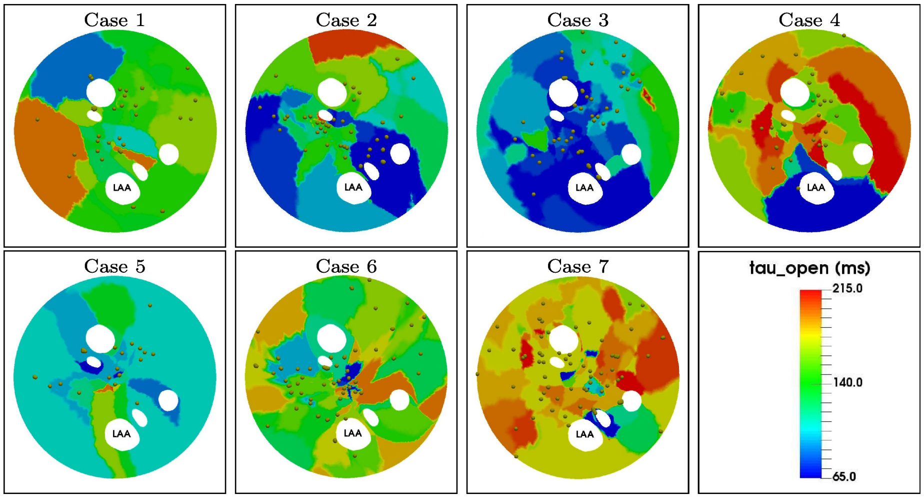

Figure 7: Distribution of τ_{open} on the atrial surface for each of the 7 cases

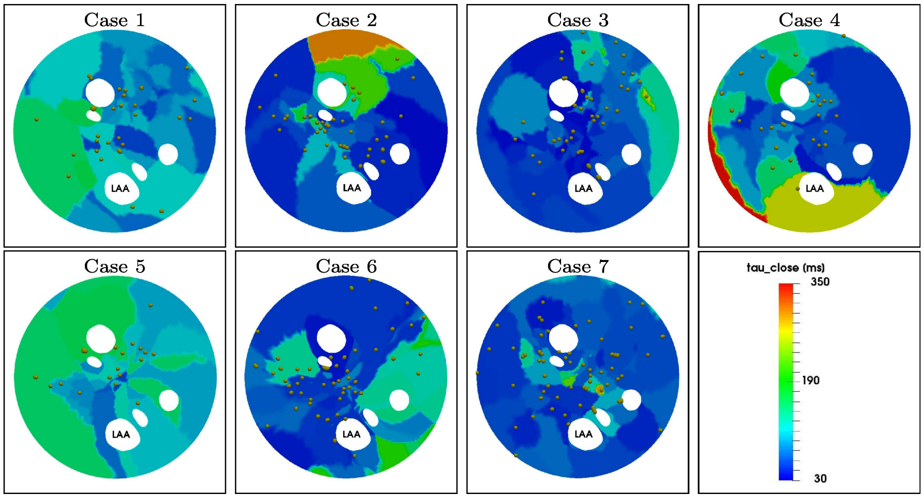

Figure 8: Distribution of τ_{close} on the atrial surface for each of the 7 cases

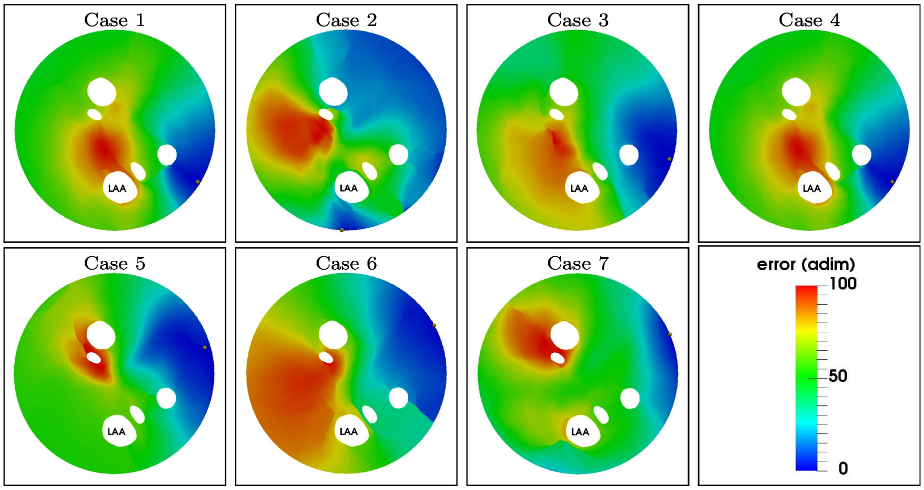

Figure 9: Dimensionless error on location of the applied stimulus; CS pacing. The gold sphere represents the location chosen to apply the stimulus on numerical simulations.

Case	1	2	3	4	5	6	7
nb of points	16248	20376	13275	18859	16972	13977	15258

Table 5: Number of points in the Velocity mesh used to reconstruct the atrial anatomy.

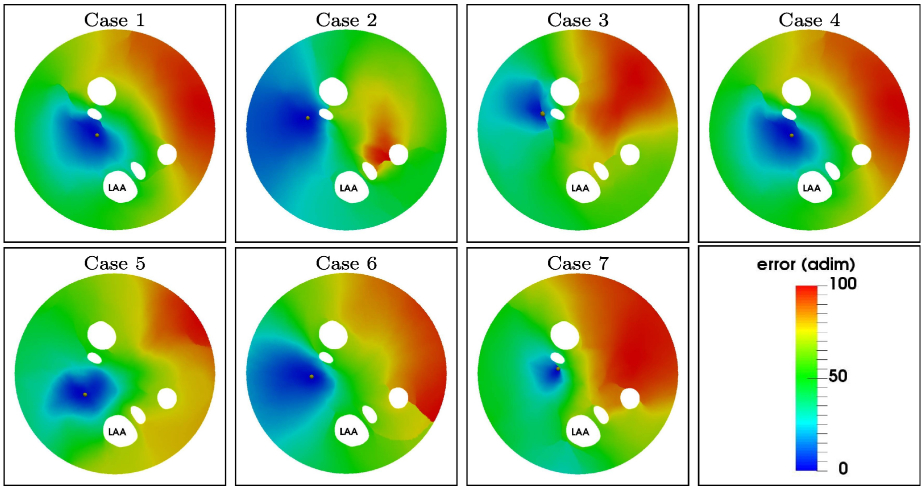

Figure 10: Dimensionless error on location of the applied stimulus; HRA pacing. The gold sphere represents the location chosen to apply the stimulus on numerical simulations.

8 Error Distribution

For each of the 7 cases, figure 11 shows the absolute error distribution on LATs for the external stimulus applied in the proximity of CS. For each of the 7 cases, figure 12 shows the absolute error distribution on LATs for the external stimulus applied at HRA.

For each of the 7 cases, figures 13 - 14 show the relative error distribution for the external stimulus applied in the proximity of CS and on the HRA, respectively.

Table 6 summarizes the mean and the standard deviation of relative and the absolute errors on LATs when the stimulus is applied either on CS or at HRA.

9 Results with different interpolation techniques

In this section we report the error on the prediction we obtained for other methodology adopted to deal with spatial parameter distribution. *Poisson equation.* Table 7 summarizes the mean and the standard deviation of relative and the absolute errors on LATs when the stimulus is applied either on CS or at

	Absolute				Relative			
	CS pacing		HRA pacing		CS pacing		HRA pacing	
Case	mean	stddev	mean	stddev	mean	stddev	mean	stddev
1	0.00	13.52	0.00	18.79	1.59	16.35	-0.47	10.93
2	0.00	8.75	0.00	20.93	2.6	11.18	-0.7	24.23
3	0.00	13.87	0.00	14.37	1.23	19.06	-0.46	16.49
4	0.00	11.55	0.00	25.79	-1.2	16.39	0.58	20.17
5	0.00	6.72	0.00	18.71	-0.98	12.99	2.38	19.49
6	0.00	15.82	0.00	16.87	-0.34	27.13	-0.03	10.21
7	0.00	21.5	0.00	12.84	1.02	23.61	0.41	11.72

Table 6: Mean and standard deviation of the absolute and the relative errors on the LATs when the electrical stimulus is applied either on CS or at HRA

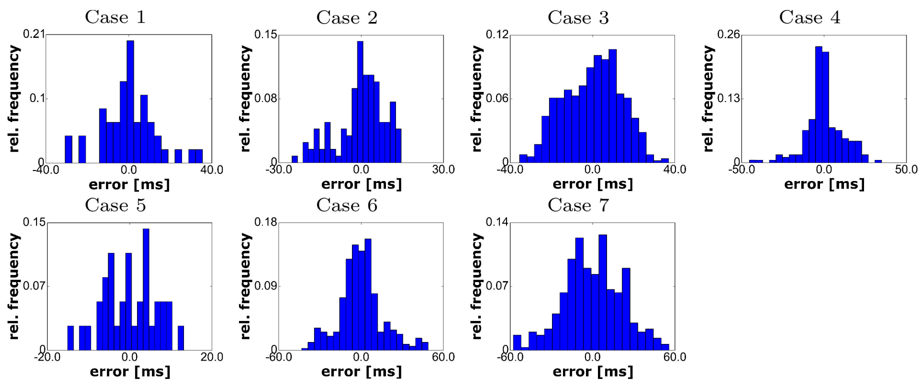

Figure 11: Absolute error distribution when the stimulus is applied on CS

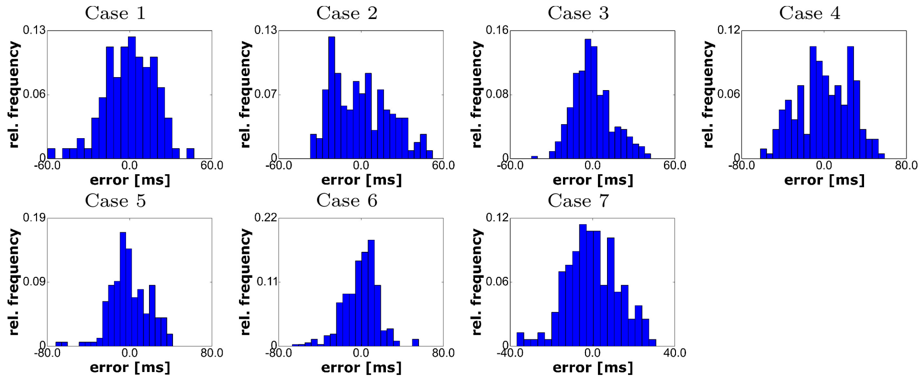

Figure 12: Absolute error distribution when the stimulus is applied at HRA

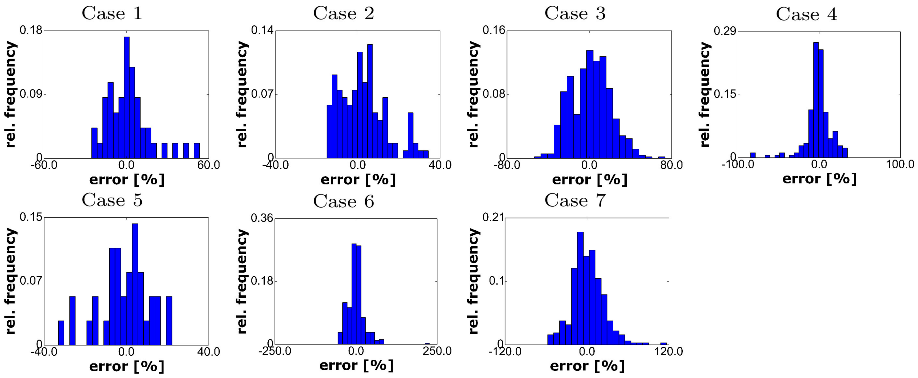

Figure 13: Relative error distribution when the stimulus is applied on CS

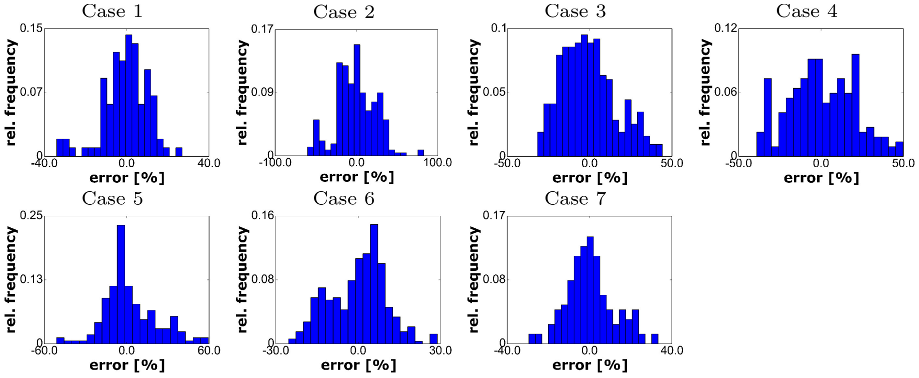

Figure 14: Relative error distribution when the stimulus is applied at HRA

	Absolute				Relative			
	CS pacing		HRA pacing		CS pacing		HRA pacing	
Case	mean	stddev	mean	stddev	mean	stddev	mean	stddev
1	0.00	15.87	0.00	13.58	1.82	17.78	0.18	8.05
2	0.00	15.97	0.00	54.85	2.19	18.77	2.56	74.46
3	0.00	13.9	0.00	14.03	1.83	23.4	-0.99	17.12
4	0.00	24.18	0.00	35.77	0.78	42.92	0.54	32.8
5	0.00	9.37	0.00	21.11	0.18	14.1	0.89	21.58
6	0.00	15.09	0.00	30.2	0.29	27.42	0.5	18.73
7	0.00	20.06	0.00	15.33	3.66	26.53	0.76	15.77

Table 7: Mean and standard deviation of the absolute and the relative errors on the LATs when the electrical stimulus is applied either on CS or at HRA and parameters are interpolated/extrapolated solving a Poisson equation.

Case	(q,m)	r	sl	fblock error	Case	(q,m)	r	sl	fblock error
1	(13.34, 0.85)	0.85	0.08	15.73%	1	(14.43, 0.92)	0.89	0.06	65.52%
2	(15.25, 0.83)	0.85	0.08	30.51%	2	(42.0, 0.59)	0.23	0.15	5.89%
3	(13.51, 0.8)	0.78	0.12	42.14%	3	(-3.9, 1.05)	0.88	0.06	2.45%
4	(6.37, 0.9)	0.61	0.19	59.27%	4	(-9.68, 1.07)	0.71	0.14	5.92%
5	(0.95, 0.99)	0.94	0.03	0.0%	5	(12.17, 0.89)	0.75	0.14	20.0%
6	(3.52 0.95)	0.86	0.07	26.98%	6	(17.45, 0.9)	0.8	0.11	0.48%
7	(21.19 0.79)	0.81	0.11	9.9%	7	(7.06, 0.95)	0.92	0.04	6.09%

Table 8: Left: Indicators used to estimate the accuracy in reproducing the experiments when a model with locally-personalised electrophysiology is adopted (CS); Right: Indicators used to estimate the accuracy in reproducing the experiments with the same model and pacing on HRA. Parameters are interpolated/extrapolated solving a Poisson equation.

HRA. Table 8 summarizes the correlations between estimated and measured LATs and the functional block errors. Figures 15 - 16 show the correlation between the computed LATs and the measured LATs for CS and HRA stimulation, respectively.

Nearest neighbours and Gaussian smoothing and variance $\sigma_G^2 = 5\text{cm}^2$. Table 9 summarizes the mean and the standard deviation of relative and the absolute errors on LATs when the stimulus is applied either on CS or at HRA. Table 10 summarizes the correlations between estimated and measured LATs and the functional block errors. Figures 17 - 18 show the correlation between the computed LATs and the measured LATs for CS and HRA stimulation, respectively.

Nearest neighbours and Gaussian smoothing and variance $\sigma_G^2 = 10\text{cm}^2$. Table 11 summarizes the mean and the standard deviation of relative and the absolute errors on LATs when the stimulus is applied either on CS or at HRA. Table 12 summarizes the correlations between estimated and measured LATs

	Absolute				Relative			
	CS pacing		HRA pacing		CS pacing		HRA pacing	
Case	mean	stddev	mean	stddev	mean	stddev	mean	stddev
1	0.0	14.78	0.0	13.65	3.59	22.07	0.06	7.44
2	0.0	36.47	0.0	23.81	8.49	54.01	-2.3	24.67
3	0.0	13.53	0.0	13.93	0.08	18.08	-1.12	17.34
4	0.0	28.44	0.0	36.14	2.07	46.79	0.58	31.14
5	0.0	9.06	0.0	22.94	0.57	14.18	1.09	22.55
6	0.0	19.32	0.0	26.46	0.5,	29.25	0.48	16.78
7	0.0	22.25	0.0	15.89	3.88	29.15	0.69	15.61

Table 9: Mean and standard deviation of the absolute and the relative errors on the LATs when the electrical stimulus is applied either on CS or at HRA and parameters are interpolated/extrapolated with nearest neighbour criterion and then smoothed with a Gaussian filter with $\sigma_G^2 = 5\text{cm}^2$

Case	(q,m)	r	sl	fblock error	Case	(q,m)	r	sl	fblock error
1	(22.39, 0.75)	0.86	0.07	10.67%	1	(5.6, 0.97)	0.93	0.04	44.83%
2	(47.61, 0.53)	0.51	0.33	6.88%	2	(-33.29, 1.33)	0.79	0.09	15.09%
3	(0.49, 0.99)	0.92	0.04	1.94%	3	(-5.12, 1.06)	0.89	0.06	0.00%
4	(12.26, 0.84)	0.64	0.19	19.45%	4	(17.66, 0.86)	0.62	0.02	4.65%
5	(4.29, 0.94)	0.94	0.03	0.0%	5	(16.62, 0.85)	0.72	0.16	12.83%
6	(4.39, 0.94)	0.84	0.08	3.84%	6	(17.07, 0.89)	0.82	0.1	4.72%
7	(20.00, 0.81)	0.8	0.11	3.02%	7	(6.06, 0.96)	0.92	0.04	0.28%

Table 10: Left: Indicators used to estimate the accuracy in reproducing the experiments when a model with locally-personalised electrophysiology is adopted (CS); Right: Indicators used to estimate the accuracy in reproducing the experiments with the same model and pacing on HRA. Parameters are interpolated/extrapolated with nearest neighbour criterion and then smoothed with a Gaussian filter with $\sigma_G^2 = 5\text{cm}^2$

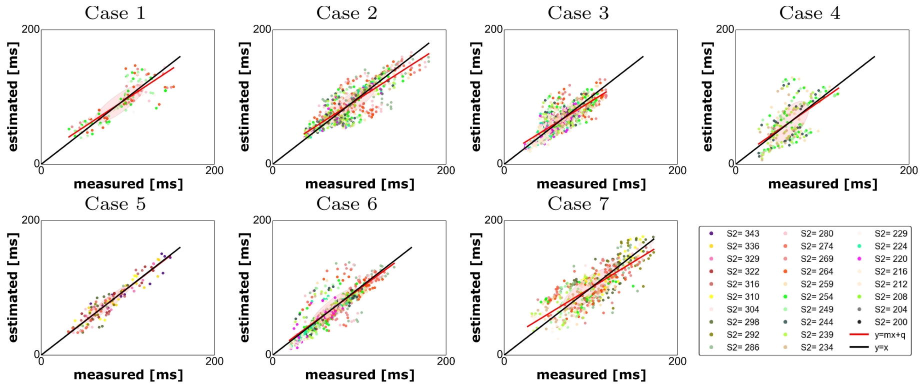

Figure 15: Measured vs estimated activation times for the personalised model (CS). Each point represents a measured vs computed LATs at each electrode and for each s_2; each colour represents the measurements taken at the electrodes for a fixed s_2 value. Parameters are interpolated/extrapolated solving a Poisson equation.

and the functional block errors. Figures 19 - 20 show the correlation between the computed LATs and the measured LATs for CS and HRA stimulation, respectively.

References

[1] C. Corrado, J. Whitaker, H. Chubb, S. Williams, M. Wright, J. Gill, M. O'Neill, and S. Niederer. Personalized models of human atrial electrophysiology derived from endocardial electrograms. *IEEE Transactions on Biomedical Engineering*, PP(99):1–1, 2016.

[2] C. Corrado, J. Whitaker, H. Chubb, S. Williams, M. Wright, J. Gill, M. O'Neill, and S. Niederer. Predicting spiral wave stability by personalized electrophysiology models. In Alan Murray, editor, *2016 Computing in Cardiology Conference (CinC)*, volume 43, pages 229–232. IEEE, Sept 2016.

[3] Cesare Corrado and Steven Niederer. A two-variable model robust to pacemaker behaviour for the dynamics of the cardiac action potential. *Mathematical Biosciences*, 281:46–54, 2016.

[4] Kotaro Fukumoto, Mohammadali Habibi, Esra Gucuk Ipek, Sohail Zahid, Irfan M. Khurram, Stefan L. Zimmerman, Vadim Zipunnikov, David Spragg, Hiroshi Ashikaga, Natalia Trayanova, Gordon F. Tomaselli, John Rickard, Joseph E. Marine, Ronald D. Berger, Hugh Calkins, and Saman Nazarian. Association of left atrial local conduction velocity with late gadolinium enhancement on cardiac magnetic resonance in patients with atrial fibrillation. *Circulation: Arrhythmia and Electrophysiology*, 9(3):e002897, 2016.

	Absolute				Relative			
	CS pacing		HRA pacing		CS pacing		HRA pacing	
Case	mean	stddev	mean	stddev	mean	stddev	mean	stddev
1	0.0	14.71	0.0	13.56	3.47	21.44	0.06	7.4
2	0.0	31.49	0.0	24.17	5.92	47.22	-2.77	25.54
3	0.0	13.73	0.0	14.06	0.22	18.39	-1.22	17.46
4	0.0	14.05	0.0	37.25	1.81	19.71	0.1	32.56
5	0.0	9.02	0.0	23.01	0.51	14.09	1.01	22.89
6	0.0	21.72	0.0	24.79	0.98	35.19	0.42	15.31
7	0.0	22.26	0.0	16.13	3.92	29.33	0.69	15.65

Table 11: Mean and standard deviation of the absolute and the relative errors on the LATs when the electrical stimulus is applied either on CS or at HRA and parameters are interpolated/extrapolated with nearest neighbour criterion and then smoothed with a Gaussian filter with $\sigma_G^2 = 10\text{cm}^2$

Case	(q,m)	r	sl	fblock error	Case	(q,m)	r	sl	fblock error
1	(21.92, 0.76)	0.86	0.07	10.67%	1	(5.75, 0.97)	0.93	0.04	44.83%
2	(35.08, 0.66)	0.62	0.23	13.16%	2	(-6.21, 1.07)	0.89	0.05	0.0%
3	(0.97, 0.99)	0.91	0.04	1.94%	3	(6.02, 0.95)	0.62	0.18	10.57%
4	(10.01, 0.89)	0.91	0.05	0.0%	4	(14.34, 0.87)	0.71	0.16	20.00%
5	(4.2, 0.95)	0.94	0.03	0.0%	5	(16.62, 0.85)	0.72	0.16	12.83%
6	(6.32, 0.92)	0.8	0.11	4.08%	6	(15.52, 0.9)	0.84	0.09	4.72%
7	(20.85, 0.8)	0.78	0.12	5.7%	7	(6.29, 0.95)	0.92	0.04	0.28%

Table 12: Left: Indicators used to estimate the accuracy in reproducing the experiments when a model with locally-personalised electrophysiology is adopted (CS); Right: Indicators used to estimate the accuracy in reproducing the experiments with the same model and pacing on HRA. Parameters are interpolated/extrapolated with nearest neighbour criterion and then smoothed with a Gaussian filter with $\sigma_G^2 = 10\text{cm}^2$

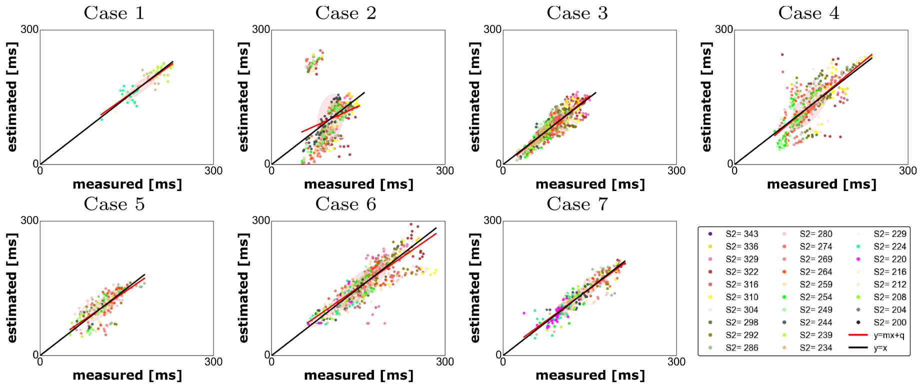

Figure 16: Measured vs estimated activation times for the personalised model (HRA). Each point represents a measured vs computed LATs at each electrode and for each s_2; each colour represents the measurements taken at the electrodes for a fixed s_2 value. Parameters are interpolated/extrapolated solving a Poisson equation.

[5] Rashed Karim, YingLiang Ma, Munjung Jang, R. James Housden, Steven E. Williams, Zhong Chen, Asghar Ataollahi, Kaspar Althoefer, C. Aldo Rinaldi, Reza Razavi, Mark D. ONeill, Tobias Schaeftter, and Kawal S. Rhode. Surface flattening of the human left atrium and proof-of-concept clinical applications. *Computerized Medical Imaging and Graphics*, 38(4):251 – 266, 2014.

[6] J. D. Murray. In *Mathematical Biology*. Springer New York, 2002.

[7] Yoshifumi Okano, Masaki Igarashi, Hideyuki Sato, Shunji Fukunaga, Kazuhiro Takamura, Kenzaburou Kobayashi, and Junichi Yamasaki. 3-d mapping of left atrial conduction pattern. *Journal of Arrhythmia*, 26(3):170 – 175, 2010.

[8] F. M. Weber, A. Luik, C. Schilling, G. Seemann, M. W. Krueger, C. Lorenz, C. Schmitt, and O. Dossel. Conduction velocity restitution of the human atrium; an efficient measurement protocol for clinical electrophysiological studies. *IEEE Transactions on Biomedical Engineering*, 58(9):2648–2655, 2011.

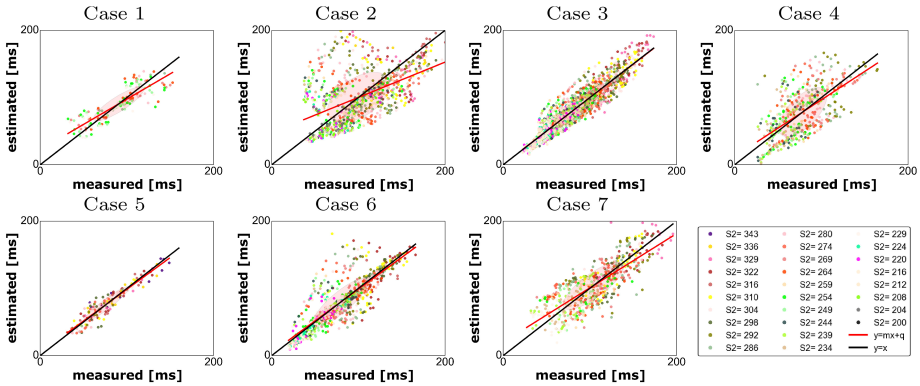

Figure 17: Measured vs estimated activation times for the personalised model (CS). Each point represents a measured vs computed LATs at each electrode and for each s_2; each colour represents the measurements taken at the electrodes for a fixed s_2 value. Parameters are interpolated/extrapolated with nearest neighbour criterion and then smoothed with a Gaussian filter with $\sigma_G^2 = 5\text{cm}^2$

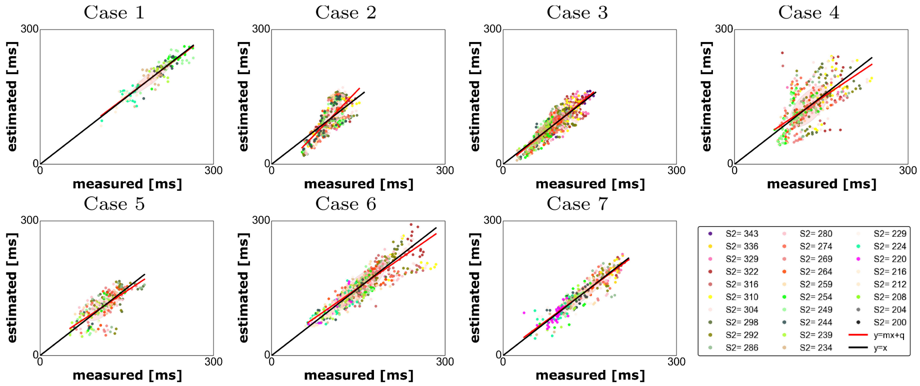

Figure 18: Measured vs estimated activation times for the personalised model (HRA). Each point represents a measured vs computed LATs at each electrode and for each s_2; each colour represents the measurements taken at the electrodes for a fixed s_2 value. Parameters are interpolated/extrapolated with nearest neighbour criterion and then smoothed with a Gaussian filter with $\sigma_G^2 = 5\text{cm}^2$

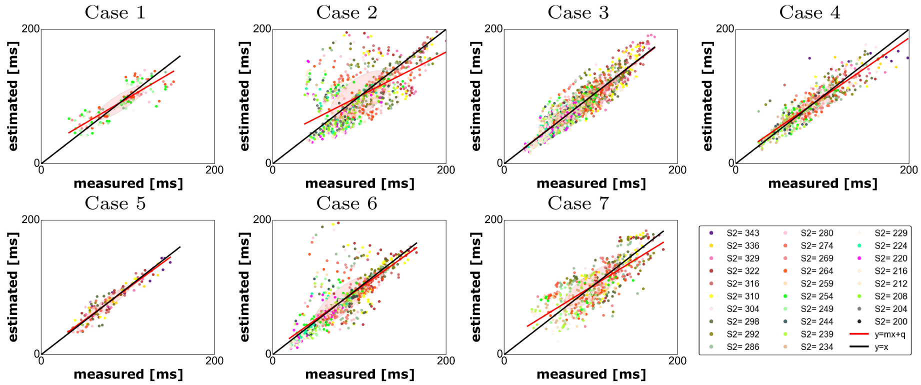

Figure 19: Measured vs estimated activation times for the personalised model (CS). Each point represents a measured vs computed LATs at each electrode and for each s_2; each colour represents the measurements taken at the electrodes for a fixed s_2 value. Parameters are interpolated/extrapolated with nearest neighbour criterion and then smoothed with a Gaussian filter with $\sigma_G^2 = 5\text{cm}^2$

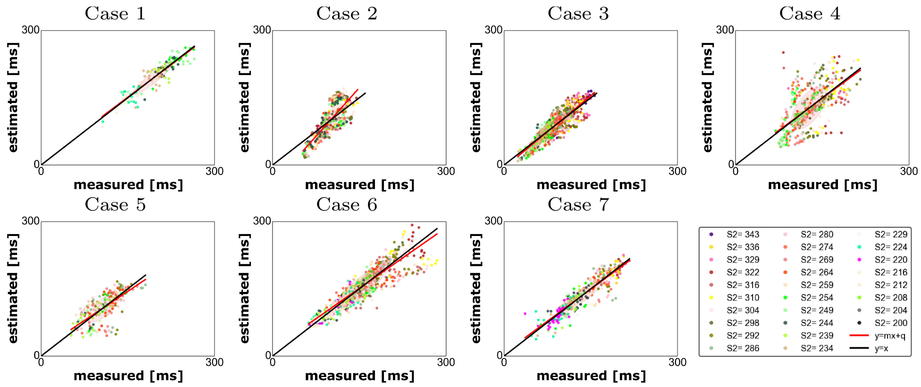

Figure 20: Measured vs estimated activation times for the personalised model (HRA). Each point represents a measured vs computed LATs at each electrode and for each s_2; each colour represents the measurements taken at the electrodes for a fixed s_2 value. Parameters are interpolated/extrapolated with nearest neighbour criterion and then smoothed with a Gaussian filter with $\sigma_G^2 = 5\text{cm}^2$
